# Systematic sequence engineering enhances the induction strength of the glucose-regulated *GTH1* promoter of *Komagataella phaffii*

**DOI:** 10.1093/nar/gkad752

**Published:** 2023-10-04

**Authors:** Mirelle Flores-Villegas, Corinna Rebnegger, Viktoria Kowarz, Roland Prielhofer, Diethard Mattanovich, Brigitte Gasser

**Affiliations:** CD-Laboratory for Growth-decoupled Protein Production in Yeast at Department of Biotechnology, University of Natural Resources and Life Sciences (BOKU), Vienna, Austria; University of Natural Resources and Life Sciences Vienna (BOKU), Department of Biotechnology, Institute of Microbiology and Microbial Biotechnology, Muthgasse 18, 1190 Vienna, Austria; CD-Laboratory for Growth-decoupled Protein Production in Yeast at Department of Biotechnology, University of Natural Resources and Life Sciences (BOKU), Vienna, Austria; University of Natural Resources and Life Sciences Vienna (BOKU), Department of Biotechnology, Institute of Microbiology and Microbial Biotechnology, Muthgasse 18, 1190 Vienna, Austria; ACIB GmbH, Muthgasse 11, 1190 Vienna, Austria; CD-Laboratory for Growth-decoupled Protein Production in Yeast at Department of Biotechnology, University of Natural Resources and Life Sciences (BOKU), Vienna, Austria; University of Natural Resources and Life Sciences Vienna (BOKU), Department of Biotechnology, Institute of Microbiology and Microbial Biotechnology, Muthgasse 18, 1190 Vienna, Austria; University of Natural Resources and Life Sciences Vienna (BOKU), Department of Biotechnology, Institute of Microbiology and Microbial Biotechnology, Muthgasse 18, 1190 Vienna, Austria; CD-Laboratory for Growth-decoupled Protein Production in Yeast at Department of Biotechnology, University of Natural Resources and Life Sciences (BOKU), Vienna, Austria; University of Natural Resources and Life Sciences Vienna (BOKU), Department of Biotechnology, Institute of Microbiology and Microbial Biotechnology, Muthgasse 18, 1190 Vienna, Austria; ACIB GmbH, Muthgasse 11, 1190 Vienna, Austria; CD-Laboratory for Growth-decoupled Protein Production in Yeast at Department of Biotechnology, University of Natural Resources and Life Sciences (BOKU), Vienna, Austria; University of Natural Resources and Life Sciences Vienna (BOKU), Department of Biotechnology, Institute of Microbiology and Microbial Biotechnology, Muthgasse 18, 1190 Vienna, Austria; ACIB GmbH, Muthgasse 11, 1190 Vienna, Austria

## Abstract

The promoter of the high-affinity glucose transporter Gth1 (P*_GTH1_*) is tightly repressed on glucose and glycerol surplus, and strongly induced in glucose-limitation, thus enabling regulated methanol-free production processes in the yeast production host *Komagataella phaffii*. To further improve this promoter, an intertwined approach of nucleotide diversification through random and rational engineering was pursued. Random mutagenesis and fluorescence activated cell sorting of P*_GTH1_* yielded five variants with enhanced induction strength. Reverse engineering of individual point mutations found in the improved variants identified two single point mutations with synergistic action. Sequential deletions revealed the key promoter segments for induction and repression properties, respectively. Combination of the single point mutations and the amplification of key promoter segments led to a library of novel promoter variants with up to 3-fold higher activity. Unexpectedly, the effect of gaining or losing a certain transcription factor binding site (TFBS) was highly dependent on its context within the promoter. Finally, the applicability of the novel promoter variants for biotechnological production was proven for the secretion of different recombinant model proteins in fed batch cultivation, where they clearly outperformed their ancestors. In addition to advancing the toolbox for recombinant protein production and metabolic engineering of *K. phaffii*, we discovered single nucleotide positions and correspondingly affected TFBS that distinguish between glycerol- and glucose-mediated repression of the native promoter.

## Graphical Abstract

**Graphical Abstract:**
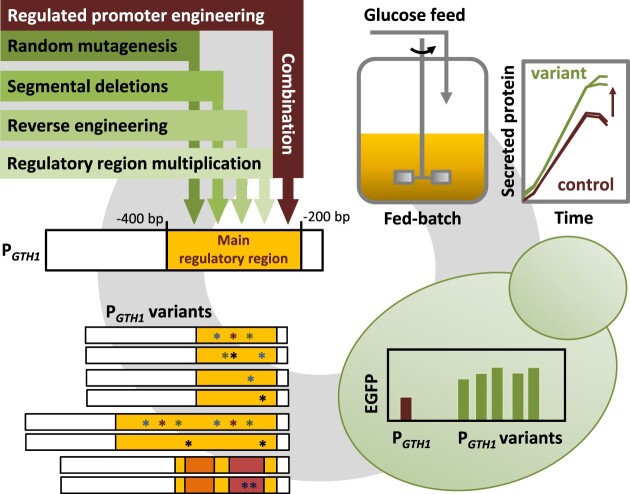

## Introduction

Promoter sequences have a fundamental role in biotechnological applications, as they determine expression strength and regulation of the recombinant gene(s). Eukaryotic promoter sequences have a core promoter, and regulatory sequences which can even be found far upstream in so-called distal elements. The core promoter element contains the region for RNA polymerase II to bind and constitutes the minimal information necessary for transcription to occur (1). Transcriptional regulation is conveyed through the binding of transcription factors (TFs) at specific transcription factor binding sites (TFBS) present in the upstream promoter regions. These TFs can act as repressors or activators of transcription, and their dynamic binding will determine the induction and repression characteristics of each gene (2). Promoter engineering studies have been on the rise in recent years to expand the repertoire of strong promoters and/or alter the induction properties of already established promoters (2–6). Traditional promoter engineering methods include random approaches such as random mutagenesis by error prone PCR (EP-PCR) (3) as well as more rational approaches, for instance, construction of hybrid promoters by combining different upstream activating sequences UAS with core promoters (1,7–11), or modifying specific sequences like TFBS or poly dA:dT stretches (12–14). Importantly, for all rational approaches, some prior knowledge of the respective promoter architecture is required. Especially hybrid promoter designs proved to be highly effective to generate novel promoters with improved strength and/or regulation, while nucleotide diversification in natural promoters by random mutagenesis often resulted in weaker promoters as TFBS are lost or TF-interactions are weakened (15). Nevertheless, random mutagenesis with subsequent cell sorting was successfully used to generate promoter libraries spanning wide ranges of expression strengths (reviewed recently in (5)). However, so far, approaches based on random mutagenesis were mostly used for constitutive (3,16–18), but only rarely for inducible promoter systems (19,20).

The methylotrophic yeast *Komagataella phaffii* (also known as *Pichia pastoris*) is widely used for recombinant protein production due to its capacity to grow to high densities and its ability to perform post-translational modifications, such as disulphide bond formation or glycosylation (21–25). The promoter of its alcohol oxidase I gene (P*_AOX1_*) is strongly induced by methanol, making it the most applied regulatable promoter for controlling recombinant protein production in this yeast (26–28). However, utilization of methanol as an inducer has some disadvantages, as it is flammable and toxic. Furthermore, alcohol oxidase requires oxygen as an electron acceptor, which leads to a high oxygen demand and heat generation in methanol-based industrial production processes (29,30). Therefore, much effort was put into establishing an array of various promoters that are methanol-independent. For constitutive expression, the promoter of glyceraldehyde 3-phosphate dehydrogenase (P_GAP_) is mostly used (31,32), while inducible promoters independent of methanol were scarce until rhamnose-inducible P*_RHAx_* (33), ethanol-regulated P*_ADH_* (34) and a collection of glucose-regulated promoters (35) were discovered.

Engineering of P_GAP_ and P*_AOX1_* gave rise to promoters with increased expression strength and/or altered induction properties. For the constitutive GAP promoter, random mutagenesis and subsequent cell sorting were applied with the aim to increase its expression strength (17). Duplication or deletion of certain TFBS present in the P_GAP_ sequence also led to higher constitutive expression levels, especially when the Gal4-like TF Cra1 was overexpressed (36). Many more studies have been dedicated to improving or altering P*_AOX1_* properties, by e.g. applying random mutagenesis (20), deletion or addition of TFBS (37), modifying the poly dA:dT regions (38), and a systematic mutagenesis of its core promoter (39). Several of these strategies did not only result in P*_AOX1_* variants with improved expression strength, but also changed the induction/repression properties of the promoter, thus allowing for methanol-free expression strategies (40,41). Targeted exchange of specific TFBS even allowed to create a synthetic P*_AOX1_* variant which can be induced by ethanol instead of methanol (42).

Such extensive promoter engineering studies have not yet been performed for the glucose-responsive promoter of the high affinity glucose transporter Gth1 (P*_GTH1_*) of *K. phaffii* (35). This promoter is repressed in glycerol and glucose excess conditions and strongly induced under glucose-limiting conditions, effectively allowing for inducible, methanol-free production processes (35,43).

P*_GTH1_* contains around 110 TFBS of 45 different families. Due to this extensive arrangement of TFBSs, rational engineering of certain binding sites was shown before as unsuited to modify the expression control of P*_GTH1_* (43). For this reason, an unbiased strategy for the enrichment and selection of improved promoter variants that simultaneously allowed to study promoter function in more depth was used in this study. Error-prone PCR (EP-PCR) based random mutagenesis of the main regulatory region of P*_GTH1_* and subsequent fluorescence activated cell sorting (FACS) in inducing and repressing conditions were performed to enrich for promoters with enhanced expression strength and largely unaltered regulatory properties. Combining data from this random approach with data from a rational approach employing segmental deletions led us to a deeper understanding of promoter regulation of P*_GTH1_*. This knowledge was then further applied to systematically reverse engineer P*_GTH1_* and generate rationally designed promoter variants that clearly outperformed their ancestors for the production of different secreted proteins in glucose-limiting production processes.

## Materials and methods

### Strains and media

*Escherichia coli* DH10B (Invitrogen) and backbone BB3aZ_14* were used for subcloning (44). BB3aZ_14* contains a Zeocin resistance cassette and the *AOX1* transcription terminator region as homologous integration locus. Prior to transformation into *K. phaffii*, all plasmids were linearized with *AscI* within the integration region in order to direct the expression vector to the respective locus. If not stated otherwise, 1 μg of plasmid DNA was used for transformation.

*K. phaffii* X-33 (Invitrogen) was used for the sorting experiment and the *K. phaffii* type strain CBS2612 (CBS-KNAW Fungal Biodiversity Centre, Centraalbureau voor Schimmelcultures, Utrecht, The Netherlands) was employed as a background strain for all other experiments in this study. Routine strain propagation and transformation were performed as described in Gasser et al. (45). YPD was prepared (per liter) with 20 g of soy peptone, 10 g of yeast extract, 20 g of glucose and 20 g of agar–agar in case of solid media.

### Error-prone PCR and construction of the promoter mutant library

Error-prone PCR (EP-PCR) was done on the −457 to −137 bp promoter region of *GTH1* (PAS_chr1–3_0011; (35)) in three consecutive steps employing primers PE1 and PE2 (Supplementary Table S1 in Supplementary File 1). Vector pPuzzle containing the P*_GTH1_*-EGFP expression cassette (35) served as the primary template. EP-PCR reactions contained 0.5 mM MnCl_2_, 10.15 mM MgCl_2_, 1.8 mM dCTP and dTTP, 0.2 mM dATP and dGTP, 0.3 μM of each primer, 20 ng template DNA, 5.0 U of FIREPol® Taq DNA polymerase (Solis Biodyne) as well as the appropriate amount of the corresponding 10-fold PCR buffer (Solis Biodyne). The gel-purified EP-PCR products of each step as well as the respective pPuzzle vector were digested with BglII and BstXI and the EP-PCR products were separately re-inserted and cloned into electrocompetent *E. coli* 10-beta (New England Biolabs). Subsequently, the plasmid libraries of each step were mixed in an equimolar manner, and transformed into *K. phaffii* X-33 (Invitrogen) according to Gasser et al. (45). To avoid multicopy integration, the amount of plasmid per transformation was lowered from 1000 to 100 ng. After regeneration in YPD at 28°C for 3 h, the transformed cells were pooled, added to 250 ml liquid YPD containing 10 μg ml^−1^ Zeocin and incubated for 48 h at 180 rpm and 25°C. From this culture, 0.9 ml aliquots were mixed with 0.1 mL of pure glycerol in a cryo-vial and stored until further use at −80°C. To estimate the library size, aliquots of the pooled transformants were plated on YPD agar plates containing 25 μg ml^−1^ Zeocin and CFUs were counted after incubation at 30°C for 48 h. To determine the error rate of the generated P*_GTH1_*_-mut_ libraries in *E. coli* at least 12, and for *K. phaffii*, 56 randomly selected clones were analysed by colony PCR employing primers PE3 and PE4 (Supplementary Table S1 in Supplementary File 1) and subsequent Sanger sequencing of the amplified promoter region. The same procedure was used to pre-screen clones for multi-copy integration and to determine the changes to the nucleotide sequence in the isolated variants.

### Sorting of the P*_GTH1_*_-mut_ library

Pre-cultures for all sorting steps were cultivated in YP media containing 10 μg ml^−1^ Zeocin at 25°C at 180 rpm overnight. Corresponding to the respective sorting strategy, main cultures were either cultivated in limiting glucose (X) or glycerol excess (G) growth conditions. To establish limiting glucose conditions, ASM synthetic media (46) containing 25 g l^−1^ polysaccharide (m2p-labs) was inoculated to a starting OD_600_ of 5.0. A glucose-release rate of 0.5 mg g^−1^ h^−1^ was established by adding the glucose-releasing enzyme (m2p-labs) to a final concentration of 0.8%. Glycerol excess conditions were realised by inoculating ASM media containing 2 g l^−1^ glycerol to a starting OD_600_ of 0.1. Both types of cultures were incubated for about 20 h at 25°C and 180 rpm. Prior to fluorescence activated cell sorting (FACS), cells were washed once (5000 rpm, 5 min, RT), resuspended in 1.0 ml phosphate-buffered saline (PBS), sonicated at 85% for 6 s (Ultrasonic processor UIS250L & Sonotrode LTS24d10.4L2, Hielscher) and diluted to a final OD_600_ of 1.0 with PBS. For FACS, a MoFlo® Astrios™ (Beckman Coulter) was used. Cells were sorted according to their EGFP fluorescence. For enrichment sortings, cells were collected in 5 ml YPD containing 100 units ml^−1^ of penicillin as well as 100 μg ml^−1^ streptomycin (Gibco™) and regenerated overnight at 25°C and 180 rpm. Single cells were directly sorted onto YPD agar plates containing 100 units ml^−1^ of penicillin as well as 100 μg ml^−1^ streptomycin (Gibco™) and incubated for 48 h at 30°C.

### Small-scale screening to analyse reporter gene expression

To analyse the expression strengths of the isolated clones from the FACS procedure as well as the effect of the rational and combinatorial promoter engineering, small-scale screenings were performed in the 24-deep-well (DWP) format. For precultures, 2 mL YP media containing the appropriate concentration of Zeocin were inoculated and incubated at 25°C under continuous shaking at 280 rpm overnight. Subsequently, the cells were centrifuged and resuspended in 1.0 ml ASM media and used to inoculate the main cultures, applying either inducing (limiting glucose) or repressing (excess glycerol) growth conditions. Inducing conditions were established by supplementing the ASM media with 50 g l^−1^ polysaccharide (m2p-labs in case of initial screening of the sorting variants and EnPresso® Y Defined for all other screenings). A defined glucose release rate of approx. 0.7 mg g^−1^ h^−1^ was established by the addition of 1.5% (m2p-labs) or 0.4% (EnPresso®) glucose-releasing enzyme. To establish repressing conditions, 2 g l^−1^ of glycerol was initially provided as a carbon source and added again after 24 h of cultivation. The starting OD_600_ for glucose-limiting conditions was 5.0, while it was 0.5 when promoter repression on excess glycerol was desired. After incubation at 25°C at 280 rpm for 48 h, intracellular EGFP fluorescence was analysed by flow cytometry as described below. Screenings for determination of secreted protein levels were conducted as described above with the exception that precultures were done on YP supplemented with 1 g l^−1^ glycerol and main cultures (inducing conditions only) were started at an OD_600_ of 8.0. On each 24-DWP plate, at least four clones of the respective control were cultivated along with six to ten clones of the various promoter variants.

### Determination of intracellular EGFP with flow cytometry

For flow cytometer analysis, cells were diluted in 96-well-plates to an OD_600_ of approximately 0.4 in PBS. Flow cytometry was performed on a CytoFLEX S (Beckman Coulter) measuring at least 10 000 events per analysis using the 525/40 nm filter. The specific fluorescence of EGFP was determined by relating it to the cell volume as described in Hohenblum *et al.* (47). The effect of each variant was calculated by comparing its fluorescence to mean values of the appropriate controls, P*_GTH1_*-EGFP or D-P*_GTH1_*-EGFP.

### Quantification of secreted recombinant proteins by microfluidic capillary electrophoresis

The ‘LabChip GX/GXII System’ (PerkinElmer) was employed for quantitative analysis of secreted protein titer (HSA or scFv; see below) in culture supernatants. The consumables ‘Protein Express Lab Chip’ (760499, PerkinElmer) and ‘Protein Express Reagent Kit’ (CLS960008, PerkinElmer) were used. Briefly, 6 μl of culture supernatant were mixed with 21 μl of non-reducing sample buffer. This mixture was denatured at 100°C for 5 min, briefly centrifuged and further mixed with 105 μl water (Milli-Q® or equivalent). Samples were then centrifuged at 1200 *g* for 2 min and applied to the instrument. Internal standards enabled for approximate allocations to size in kDa and to determine concentrations from the detected signals. Afterwards, the concentration of the secreted recombinant protein (titer) was related to the wet cell weight (WCW) to determine the product yield (mg product per g WCW). For all screenings, protein titers and yields are given as fold change (FC) relative to the average of the four reference clones cultivated on the same 24-DWP.

### Statistical analysis of screenings

Outlier identification of screening data was done using the modified *Z* score method with an absolute *Z* score threshold of 3.5 (48). Subsequently, statistical differences between promoter variants and the respective controls cultivated in the same screening round were analysed by Student's t-test.

### Cloning strategies for promoter engineering

#### P_GS_*_n_* variants

To verify that the EP-PCR based mutations found in the promoter variants isolated by the sorting procedure (P*_GTH1_*_-mut_) are indeed responsible for the observed differences in expression strength, the respective promoter sequences were amplified along the other parts of the EGFP expression cassette from genomic DNA employing primers PE5 and PE6 (Supplementary Table S1 in Supplementary File 1) and cloned into BB3aZ_14*. The corresponding promoter variants were named P_GS_*_n_*.

#### Point mutations promoter variants

Were constructed by PCR employing a pair of overlapping primers which both contained the desired mutation (PE7 to PE26; Supplementary Table S1 in Supplementary File 1) as well as plasmid BB3aZ-P*_GTH1_*-GFP as a template. The purified PCR product was then digested with DpnI for 1 h and transformed into chemically competent *E. coli* DH10B (Liu & Naismith, 2008). The corresponding variants were named P_GMutX_.

#### Duplication of the main regulatory region of P*_GTH1_*variants

Duplicating the −126 bp to −506 bp region of the different variants was done by assembling 3 PCR fragments. The three fragments were amplified using primers PE5 and PE27 for the end part of the promoter (first fragment; −507 to −965 bp), PE28 and PE29 for the duplicated part (second fragment; −126 to −506 bp) and PE30 and PE6 for the third fragment (−506 bp to the end of the GFP gene; Supplementary Table S1 in Supplementary File 1). Subsequently, the three fragments were cloned into BB3aZ_14* by Golden Gate assembly. To indicate the duplication of the main regulatory region the prefix ‘D-’ was added to the name of the respective promoter variant.

#### Sliding window deletions

The region −200 to −400 bp of P*_GTH1_* was studied further by constructing 10 different promoter variants of which 30 bp were deleted in an overlapping manner, so that 10 bp of the deleted region were shared with the respective adjacent variants. These sliding window deletions were generated by amplifying plasmid BB3aZ-P*_GTH1_*-GFP with primer pairs that bind outside of the region to be removed (primers PE31 to PE50; Supplementary Table S1 in Supplementary File 1). Subsequently, the ends of the PCR amplicon were joined via Golden Gate cloning.

#### Sequential multiplications

Based on the results of the sliding window deletions, we chose two regions to be duplicated: a) the region that corresponds to deletions 2 and 3 excluding the parts shared with deletion 1 and deletion 4, as well as b) the region corresponding to deletions 5–7 without the parts shared with deletion 4 and deletion 8. Duplication of these regions was done by joining two PCR fragments which both contained the segments to be duplicated. The segments were amplified using primers PE51 and PE52 or PE53 and PE54 (Supplementary Table S1 in Supplementary File 1) in combination with primers PE5 and PE6, respectively. Plasmid BB3aZ-P*_GTH1_*-GFP was used as a template. The two fragments were cloned into BB3aZ_14* via Golden Gate cloning. The resulting promoter variants were named P_GDup2-3_ and P_GDup5-7_. Additionally, a variant that contained three copies of region Del2-3 (P_GTrip2-3_) was constructed employing primers PE51 and PE52 with PE5 and PE6 (Supplementary Table S1 in Supplementary File 1) using the plasmid containing the P_GDup2-3_ variant as a template.

#### Promoter variants in combination with secreted model proteins

To generate the plasmids for the recombinant secretory reporters, the EGFP coding sequence in the plasmids containing the promoter variants was either exchanged for the coding sequence of human serum albumin (HSA, with its native secretion leader) or a single chain variable fragment (scFv) via classical cloning employing the restriction enzymes SbfI and SfiI (both New England Biolabs). Secretion of scFv was driven by the *S. cerevisiae* alpha mating factor secretion leader. The respective donor plasmids are described in Prielhofer *et al.* (35) for HSA and in Zahrl *et al.* (49) for scFv.

### *In-silico* TFBS analysis

Analysis of the TFBS of the native *GTH1* promoter as well as the promoter variants designed in this study was done by MatInspector (50), using the MatInspector library version 11.1 with the fungi matrix as reference. The parameters were set as default and the similarity scores higher than 0.75 were considered as potentially true matches. To determine the differences of the promoter variants the Common TF package was used with the same settings.

### *In-silico* mutations

To design single point mutation variants that affected certain TFBS, we performed an *in-silico* mutational analysis of the *GTH1* promoter region that was densely packed with TFBS. Using a MATLAB script, each of the 740 nucleotides upstream of the *GTH1* gene was computationally exchanged for each of the other 3 nucleotides. Exemplarily, if the original position was an A, we exchanged it to either T, C or G. The resulting 2220 promoter variants, each containing a single nucleotide exchange, were put in sets of 48 and analysed by MatInspector as described previously to determine which single mutation led to a deletion or an addition of one or more TFBS.

### Repression experiment

To gain a deeper understanding of the regulatory properties of variants P_GMutBPDup2-3_ and D-P_GMutBP_ in respect to the provided carbon source, a repression experiment was conducted. Pre-cultures of the respective EGFP-expressing strains were grown on YP media for 24 h at 28°C. For main cultures, ASM media containing either glycerol or glucose was inoculated to a final OD_600_ of 0.5. Three different concentrations of the respective carbon source were added to the media (1, 2 and 4%). After 8, 24 and 48 h the cells were resuspended in PBS and EGFP fluorescence was measured on a flow cytometer as described above.

### Determination of gene copy number and transcript levels by quantitative real-time PCR (qPCR)

To determine the gene copy number of the respective recombinant reporter gene, genomic DNA was isolated from cells pelleted from 1 mL of an overnight YPD culture (180 rpm, 25°C) employing the Wizard® Genomic DNA Purification Kit (Promega). The resulting genomic DNA concentration and purity was measured with a Nanodrop 2000 (Thermo Scientific™).

For transcript level determination RNA was isolated from fed-batch samples by using TRI reagent according to the supplier's instructions (Ambion, USA). To remove residual DNA, the RNA samples were treated with the DNA-free™-kit (Ambion) according to the manufacturers’ manual. Subsequently, RNA quality, purity and concentration were analysed by gel electrophoresis as well as spectrophotometric analysis using a NanoDrop 2000 (Thermo Scientific). Synthesis of cDNA was done with the Biozym cDNA Synthesis Kit according to the manufacturer′s manual. Instead of the provided primers, Oligo d(T)23 VN primer (NEB) was used.

For qPCR analysis appropriate amounts of the cDNA (for transcript level analysis) or genomic DNA (for gene copy number analysis) were mixed with water, primers and 2x qPCR S’Green BlueMix (Biozym Blue S’Green qPCR Kit) according to the supplier's recommendations and measured in a real-time PCR cycler (Rotor-Gene, Qiagen). All samples were measured at least in technical triplicates. Data analysis was performed with the Rotor-Gene software employing the Comparative Quantitation (QC) method, using *ACT1* as reference gene. Supplementary Table S1 in Supplementary File 1 provides an overview of the primers used for qPCR (PE55–PE66).

### Fed batch cultivation

Fed-batch cultivations were performed as described before for P*_GTH1_*-*D1240* (43). For pre-cultures, 100 ml YPD media containing 50 μg l^−1^ Zeocin in a 1 l shake flask were inoculated with a 1.0 ml cryostock and incubated for approximately 24 h at 180 rpm and 25°C. Batch cultures were operated in 1 l DASGIP benchtop bioreactors (SR0700ODLS; Eppendorf AG) at a working volume of 0.25 l and were inoculated to a starting OD_600_ of 1.5. The cultivation temperature was 30°C. The dissolved oxygen was kept at 30% by automated adjustment of stirrer speed and air flow, and the pH was regulated at the appropriate setpoint for the respective recombinant protein by automated addition of 12.5% NH_4_OH. After a sudden spike in DO indicating batch-end, a linear incremental glucose feed resulting in fast initial growth rates followed by an extended phase of gradually decreasing specific growth rate was applied (43).

Yeast dry mass (YDM) and secreted recombinant protein levels were analysed throughout the process. For YDM analysis, 1 ml of culture broth was transferred to a 2 ml centrifugation tube, which was pre-dried at 105°C for at least 24 h and pre-weighted. After centrifugation at 16 000 *g* for 5 min the supernatant was transferred to a fresh vial and stored at −20°C until further use. Cell pellets were washed twice with deionized water and dried at 105°C for at least 24 h before the weight was measured again. Supernatants were analysed by microfluidic capillary electrophoresis (GXII, Perkin-Elmer) as described above.

## Results

### EP-PCR-based random mutagenesis and P*_GTH1_*_-mut_ library construction

**Figure 1.**
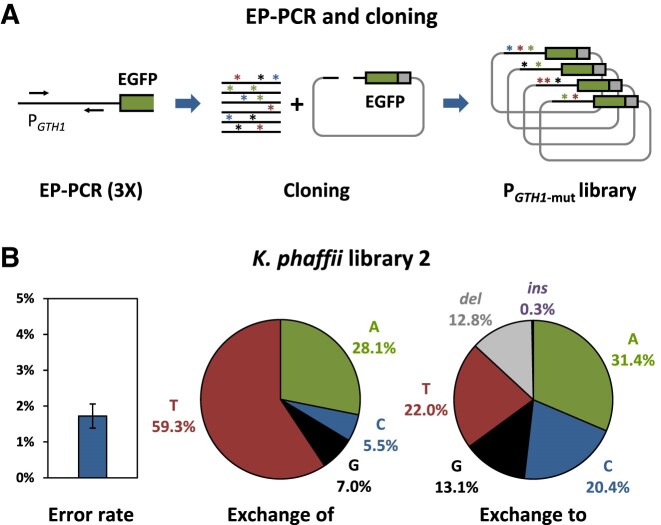
Schematic workflow of the random mutagenesis approach leading to the *K. phaffii GTH1* promoter library. (**A**) Random mutagenesis strategy: EP-PCR was conducted on the main regulatory region of P*_GTH1_* in three consecutive steps. Subsequently, the EP-PCR product was re-cloned into the appropriate position in the P*_GTH1_-*EGFP expression vector, resulting in the P*_GTH1_*_-mut_-EGFP plasmid library, which was then transformed into *K. phaffii* X33. (**B**) The average error rate and nucleotide exchange profile of the generated *K. phaffii* P*_GTH1_*_-mut_ library 2 is based on sequencing of 56 randomly selected clones. Error bars represent the 95% confidence interval.

Error-prone PCR (EP-PCR) based random mutagenesis and subsequent fluorescence activated cell sorting (FACS) in inducing and repressing conditions was performed with the aim to isolate variants with enhanced induction strength, while simultaneously attempting to preserve the repression properties of P*_GTH1_*. Based on previous findings in regard to the promoter architecture, EP-PCR-based random mutagenesis was targeted to the main regulatory region of P*_GTH1_* (from −137 to −457 bp; (43)). The main regulatory region was selected as it is highly dense of putative TFBS (Supplementary Table S2 in Supplementary File 1). By conducting EP-PCR in three consecutive steps, an increase in the average mutation rate from 1.5% at step one, to 2.7% at step two, and finally 3.4% at step three was achieved. Pooling of the isolated plasmid library from every separate step in an equimolar manner resulted in an estimated library size of 5.9 × 10^6^ promoter variants. Promoter variants created by this approach are referred to as P*_GTH1_*_-mut_. A schematic overview of the P*_GTH1_*_-mut_ library construction is shown in Figure 1A.

Initial *K. phaffii* library construction as well as FACS according to our standard protocols (‘*K. phaffii* library 1″) resulted exclusively in the isolation of high-expressing clones that harboured multiple copies of the P*_GTH1_*_-mut_-EGFP expression vector (data not shown). Hence, three key measures were taken in order to reduce multi-copy integration (‘*K. phaffii* library 2″) and lessen the enrichment of multicopy clones during the sorting procedures. First, the amount of plasmid DNA for *K. phaffii* transformation was reduced by 10-fold and second, selection for positive clones was done in liquid culture rather than on solid media, enabling to reduce the concentration of the applied selection marker by 2.5-fold. Both measures were previously demonstrated to significantly reduce multicopy integration during *K. phaffii* library construction in the work of Boone (51) and also resulted in a substantially lower multicopy frequency in this study (Supplementary Table S3 in Supporting File 1). As a final measure, the sorting gate during the first enrichment round (inducing conditions) was set in a manner as to exclude the top 1.0% (Supplementary Figure S1 in Supplementary File 1), which were likely to represent multi-copy clones. Indeed, 22 out of 30 randomly picked clones from this population were confirmed to carry multiple copies of the EGFP expression cassette, while only 3 out of 28 clones of the gated ‘Good 20’ population contained more than one EGFP copy (Supplementary Table S3 in Supplementary File 1).

*K. phaffii* library 2 was estimated to contain 6.0 × 10^4^ individual clones. The median number of mutations identified in the respective P*_GTH1_*_-mut_ variants was 5.5, whereby predominantly exchange of the nucleobases thymine and to a lesser degree adenine was observed (Figure 1B). The pattern of exchange to a certain nucleobase was more evenly distributed (Figure 1B) and 12.8% of the mutations were deletions. Insertions occurred at a much lower frequency and were observed in only one of the analysed clones.

### Isolation of P*_GTH1_* variants with improved induction strength and preserved repression properties by a tailor-made cell sorting strategy

In order to select those P*_GTH1_*_-mut_ variants from the library that showed enhanced induction strength but simultaneously retained their repression properties, FACS was conducted in four consecutive sorting rounds (Supplementary Figure S1 in Supplementary File 1). Sorting round one, three and four were done in inducing (glucose-limiting) conditions, while for sorting round two the cells were cultivated in repressing conditions (Supplementary Figure S1 in Supplementary File 1). For this particular step, cells were selected that showed low fluorescence (similar or lower fluorescence levels than the P*_GTH1_* control strain cultivated in the same repressing conditions) and therefore presumably contained variants with preserved repression properties. Single cell sorting was done in sorting round four, where 42 clones of the top 5% and, in an additional experiment, 22 clones of the top 1% were isolated for further analysis.

In addition, 30 clones were isolated from the top 1.5% of the original library under repressing (glycerol excess) conditions (Supplementary Figure S2A in Supplementary File 1) in order to shed more light on which positions/binding sites convey the repression properties to the promoter.

The isolated clones were tested for single copy integration and analysed for their induction strength in comparison to the native *GTH1* promoter. In total, 6 out of 64 analysed clones from the respective single cell sorting steps in inducing conditions proved to have a single copy of the expression vector integrated and simultaneously showed significantly increased EGFP productivities (>1.5-fold). Unexpectedly, also 7 out of the 30 clones that were selected based on their high fluorescence levels in glycerol excess showed significantly increased EGFP-expression levels (>1.5-fold) in limiting-glucose conditions (Supplementary Figure S2B in Supporting File 1).

### Verification of mutant P*_GTH1_* variants

**Figure 2.**
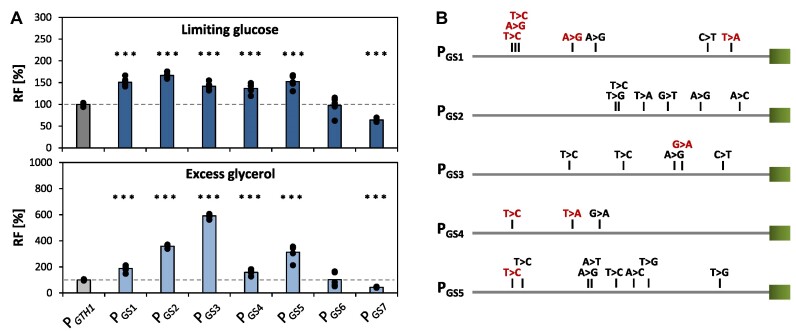
Relative EGFP fluorescence intensity and schematic representation of the identified point mutations. (**A**) Relative fluorescence levels (RF) of the top seven promoter variants re-cloned into Golden *Pi*CS and transformed into *K. phaffii* CBS2612. Six independent clones per variant were analyzed and compared to four replicates of the P*_GTH1_* control strain. Outliers were excluded from the analysis. Bars represent mean values and closed circles the calculated RF values of the individual clones. Cells were grown in the 24-DWP format in inducing (limiting glucose) and repressing (excess glycerol) conditions and EGFP levels measured on a flow cytometer. Relative fluorescence levels were calculated by normalizing to the P*_GTH1_* control. The horizontal dotted line highlights the average expression level of the native P*_GTH1_* control strain (set to 100% for each condition). Statistical analysis was done employing Student *t*-test (**P≤ 0.05; ***P≤ 0.005). (**B**) Schematic representation of the point mutations and their respective location identified in the five promoter variants for which an increased induction strength could be confirmed based on the results shown in (A). The red colored mutations are located outside of a TFBS. Further information regarding the identified mutations as well as sequence alignment of the relevant region is provided in Supplementary Table S4 and Supplementary Figure S3 in Supporting File 1.

To verify that the observed changes in the repression and induction properties of the isolated P*_GTH1_*_-mut_ variants were indeed caused by the introduced mutations rather than by clonal effects, the seven variants with the highest induction strength in the previous screenings were re-cloned into a Golden*Pi*CS vector with EGFP as reporter (44) and transformed into *K. phaffii* CBS2612. Subsequently, the expression of six individual clones per variant (called P_GS_*_n_* from here on) was tested in limiting glucose and excess glycerol conditions (Figure 2A) in the established small-scale screening format. Five of these sorting variants (P_GS1-5_) showed a significant increase in EGFP expression, exceeding the native *GTH1* promoter by 40% to 60% in inducing conditions. However, all of them were to some degree de-repressed in glycerol excess, showing between 1.6- to 6.2-fold higher EGFP levels compared to the native promoter. While this level of de-repression might appear high, overall expression levels on glycerol remained still very low and were well below those of P_GAP_. As could be expected, P_GS3_, the variant that was isolated based on its high EGFP levels in glycerol excess conditions (Supplementary Figure S2 in Supplementary File 1), showed the highest degree of de-repression. For the remaining two studied variants (P_GS6-S7_) improved EGFP expression levels from the initial screening could not be confirmed, hence, they were not further investigated.

### Mutated transcription factor binding sites in the P_GS1-5_ variants are predominantly connected to nutrient sensing and response

To predict the affected TFBS in each promoter variant, the sequences of P_GS1-5_ were subjected to bioinformatics analysis using MatInspector (Genomatix). As expected from the final mutation rate after EP-PCR, each promoter variant contained multiple point mutations (ranging from three to eight nucleotide exchanges) in the main regulatory region (Figure 2B and Supplementary Figure S3 in Supporting File 1). The majority (69%) of these mutations were located within a TFBS, and ca. 66% were transitions (Figure 2B and Supplementary Table S4 in Supporting File 1). All predicted TFBS within the region used for the EP-PCR including their positions are summarized in Supplementary Table S2.

Out of all the identified mutations, only one was shared among three out of the five promoter variants (−453T > C). This mutation was predicted to add an additional carbon source responsive element (CSRE) to the original promoter. The other point mutations showed greater diversity in respect to their location (Figure 2B). Although we did not find a specific region of the promoter that was predominantly affected by the mutations, MatInspector analysis revealed that there were some common TFBS families affected in the P_GS1-5_ variants. In addition to the F$CSRE family (binding sites for Cat8 and Sip4), also the number of F$PRES and F$MGCM sites (potentially bound by TFs Aro80, Cep3, Lys14, Rgt1, Stb4, Yrm1 and Yrr1) changed in three of the variants. In two promoter variants, the numbers of F$GATA (potentially bound by TFs Dal80, Gat1, Gln3 and Gzf3) and Y$MIG (binding site of the glucose-responsive repressors Mig1/2) were altered (Table 1). There is a common denominator in the affected TF families, as they all regulate genes in response to nutrients (either carbon source or nitrogen availability).

**Table 1.** *In-silico* prediction by MatInspector of altered TFBS in the promoter variants isolated after EP-PCR and FACS as well as those created by introducing single point mutations

Type of variant	Promoter	Mutations^a^	TFBS gained	TFBS lost
Sorting variants	P_GS1_	−453T > C, −449A > G, −445T > C, −372A > G, −340A > G, −189C > T, −151T > A;	CSRE, MGCM, YMIG;	YABF
	P_GS2_	−308T > G, −304T > C, −269T > A, −237G > T, −190A > G, −138A > C;	DUIS, PHD1, YCAT, YSTR;	FKHD, GATA, MGCM, PRES, YABF;
	P_GS3_	−379T > C, −304T > C, −235A > G, −225G > A, −167C > T;	AZF1	GATA, PDRE, PRES, YMIG, YQA1;
	P_GS4_	−453T > C, −374T > A, −336G > A;	CSRE	ASG1
	P_GS5_	−453T > C, −442T > C, −353A > G, −347A > T, −313T > C, −291A > C, −269T > G, −169T > G;	CSRE, ROX1, YABF, YGAL, YMAT, YMSE;	BZIP, HOMD, ICGG, MGCM, PRES, RDR1, RFXP, RRPE, TALE;
Point mutation variants	P_GMutA_	−445T > C	CSRE, MGCM;	
	P_GMutB_	−453T > C	CSRE	
	P_GMutC_	−320G > A	CSRE, YORE, VTBP;	
	P_GMutD_	−322A > G	CSRE	GATA, MGCM, RDR1;
	P_GMutP_	−235A > G		MIG
	P_GMutQ_	−225G > A	MGCM	MIG, PDRE, YQA1;
	P_GMutR_	−258T > A	MGCM, YORE;	MIG, ICGG, YMCM, RDNA;
	P_GMutS_	−251C > A	YMCM	MIG
	P_GMutT_	−249T > G	GAL	MIG
	P_GMutU_	−238A > G	MGCM, ARPU, YORE, ASG1	MIG
	P_GMutBP_	−453T > C, −235A > G	CSRE	MIG

^a^Position upstream of start codon and type of nucleotide exchanges in the P*_GTH1_* promoter region.

### Systematic introduction of single point mutations into the main regulatory region of P*_GTH1_* demonstrates that the positioning and genetic context of a certain TFBS is crucial to execute its transcriptional regulation

As most identified point mutations in the P_GS1-5_ variants were scattered fairly even across the mutated region, it remained unclear which specific mutations (or combinations thereof) lead to the improved properties. Hence, to gain some further insight we decided to investigate how the addition or loss of relevant TFBS (guided by point mutations) impacts the regulatory properties of P*_GTH1_*. Emphasis was given to (re-)create or delete TFBS that are connected to nutrient sensing and regulation as these emerged as the most relevant targets from the random mutagenesis approach. As the main regulatory region is fully loaded with TFBS, any sequence change could also disrupt its tight regulatory properties (43). For this reason, we replaced only single nucleotides that were predicted to change the TFBS of interest (Table 1). The respective positions were identified by MatInspector analysis of *in-silico* mutations where each nucleotide located within the relevant region was exchanged for the other three 3 nucleotides (e.g. A to T, C or G). Inevitably, even such single point mutations impacted not only the targeted TFBS, but led in many cases also to the creation or deletion of other putative TFBS according to MatInspector. A comprehensive list of the created promoter variants harboring single point mutations, their position as well as the type of exchange and the respective TFBS created or lost through these mutations is provided in Table 1.

**Figure 3.**
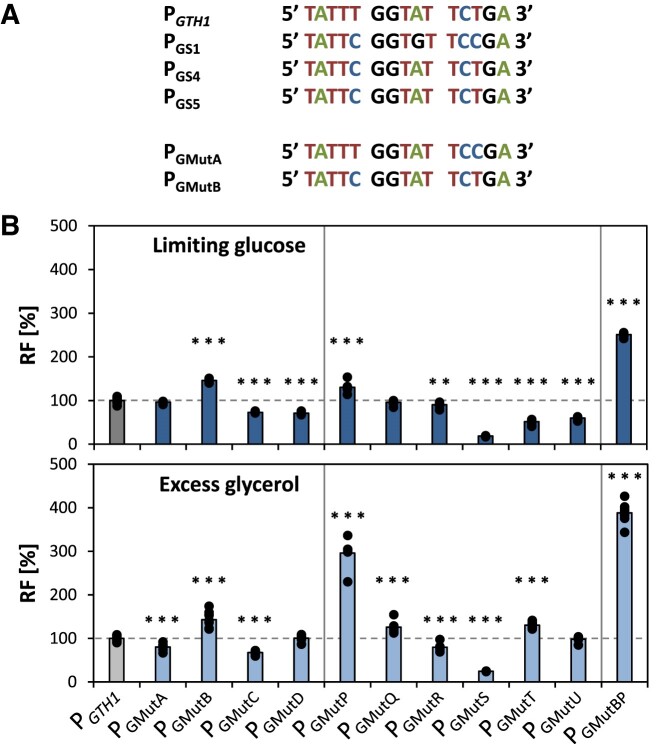
Impact of single point mutations creating additional CSRE or reducing MIG-TFBS on expression properties of P*_GTH1_*. (**A**) Sequence information on the point mutations identified in the P_GS1_, P_GS4_ and P_GS5_ variant in the −453 to −445 bp region which lead to the creation of an additional F$CSRE binding site and their recreation by point mutations MutA and MutB. (**B**) Relative EGFP fluorescence levels (RF) of at least seven independent clones per point mutation variant as compared to at least four replicates of the P*_GTH1_* control strain. Outliers were excluded from the analysis. Bars represent mean values and closed circles the calculated RF values of individual clones. Cells were cultivated in the 24-DWP screening format in either inducing (limiting glucose) or repressing (excess glycerol) conditions. Fluorescence was measured on a flow cytometer. Statistical analysis was done employing Student *t*-test (**P≤ 0.05; ***P≤ 0.005). The horizontal dotted line highlights the average expression level of native P*_GTH1_* (set to 100% for each condition). Vertical lines separate point mutations that create an additional CSRE and the deletion of one MIG site, respectively.

The shared mutation found in the sorting variants P_GS1_, P_GS4_ and P_GS5_ (−453T > C; Figure 3A) leads to the gain of one CSRE binding site. In P_GS1_ a second mutation in the vicinity of the shared mutation is present, which also leads to the gain of a CSRE TFBS. Thus, we reconstructed the point mutation that was only present in P_GS1_ (−445T > C), called P_GMutA_, and the mutation that was shared between the three variants, called P_GMutB_ (Figure 3A and Table 1). To better understand the role of the F$CSRE binding site, we created two additional single point mutation variants were one of these sites was gained (P_GMutC_ and P_GMutD_) according to the MatInspector analysis of the *in-silico* mutants. All point mutation variants were cloned upstream of EGFP and screened for their induction and repression properties in comparison to P*_GTH1_* in the established 24-DWP screening format in limiting glucose and excess glycerol conditions, respectively (Figure 3B). Out of all four point mutations that lead to the gain of an F$CSRE (P_GMutA_, P_GMutB,_ P_GMutC_ and P_GMutD_), only point mutation MutB resulted in 50% higher EGFP expression levels in limiting glucose compared to native P*_GTH1_*. Relative EGFP levels in glycerol excess conditions were approximately 20% higher. Therefore, the improved induction properties of P_GS1_, P_GS4_ and P_GS5_ might be solely explained by MutB, while the slight loss in their repression properties is probably caused by (some of) the additional mutations they contain. As shown in Table 1, introduction of the point mutations MutA, MutC and MutD affected additional TFBS families besides adding one CSRE. Indeed, P_GMutB_, which led to a 1.5-fold increase in induction strength, was the only variant in which no other TFBS besides the CSRE was gained or lost. These results indicate that not only the number but also the positioning and genetic context of a certain TFBS is crucial to execute transcriptional regulation.

Next, we focused on those point mutations that caused the loss of one Y$MIG site, as Mig1 is a TF associated with glucose repression (52), and thus deletion of the respective TFBS should in theory lead to altered induction/repression behaviour. For this purpose, we separately recreated the two point mutations present in variant P_GS3_ which led to the loss of one Y$MIG site (P_GMutP_ and P_GMutQ_, Table 1). Introduction of MutP resulted in a single deleted Y$MIG site without affecting any other TFBS. This variant presented a ca. 1.3-fold higher expression level than P*_GTH1_* in limiting glucose and also showed a high degree of de-repression in excess glycerol conditions (Figure 3B). On the other hand, the inducing properties of P_GMutQ_ remained mostly unaffected, and repression properties were only slightly decreased. Hence, MutP seems to be the main mutation responsible for the altered regulation properties of P_GS3_, which has been isolated based on its high expression in repressing conditions.

To study the importance of the Y$MIG-TFBS in more detail, we constructed further point mutation variants to decrease the number of MIG binding sites in different positions (Table 1). As for the F$CSRE variants, we used the *in-silico* mutations protocol to choose the nucleotides to be mutated to construct promoter variants that had one Y$MIG-TFBS less. In contrast to MutP, the remaining four mutations variants (P_GMutR, S, T, U_) showed either similar or even lower EGFP expression levels in limiting glucose (Figure 3B), again underlining that the genetic context of specific TFBS is highly important. Specifically, MutS resulted in a very strong decrease in EGFP expression in inducing as well as repressing conditions, suggesting that this particular position is highly important for promoter function.

Another TFBS family that was affected in several of the sorting variants was F$MGCM (monomeric Gal4-class motifs), however, for this family both, the gain (P_GS1_) as well as the loss (P_GS2_, P_GS5_) of a MGCM site were observed. Seemingly, the MGCM sites are closely related to other TFBS, as five of the point mutations introduced to create an additional CSRE- or remove a MIG-TFBS simultaneously led to the gain (P_GMutA_, P_GMutQ,_ P_GMutR_ and P_GMutU_) or loss (P_GMutD_) of a MGCM site. As seen before, the variants P_GMutA_, P_GMutQ_ and P_GMutR_ did not show any change in their inducing properties, while MutD and MutU even led to a decrease in induction strength. Hence, it seems that the number of F$MGCM binding sites does not correlate with the induction or repression properties of P*_GTH1_* and its variants.

Notably, the two single point mutations which caused the highest increase in promoter activity in inducing conditions (P_GMutB_ and P_GMutP_) were also found in some of the sorting variants, and were the only ones that only affected the targeted TFBS when being reverse engineered. In a next attempt, a novel promoter variant was created by combining both mutations (Supplementary Figure S4 in Supplementary File 1). This double point mutation variant (P_GMutBP_) showed a 2.5-fold increase in EGFP expression levels in inducing conditions (Figure 3B), which was much higher than what was achieved with any of the single point mutation variants individually (1.6- and 1.3-fold, respectively), indicating that the effect of both point mutations is synergistic.

### Duplication of the most important promoter region enforces the regulatory properties of the promoter variants

**Figure 4.**
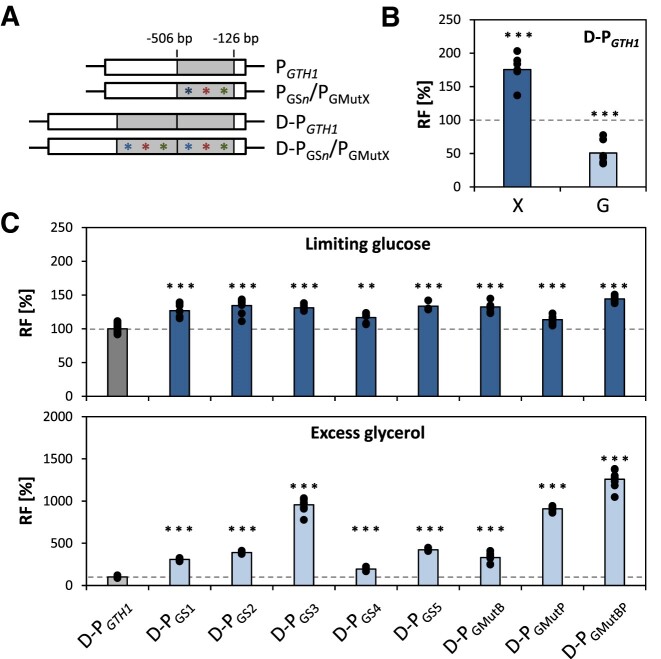
Duplication of the main regulatory promoter region increases induction strengths of all promoter variants. (**A**) Schematic representation of the duplication of the main regulatory region of P*_GTH1_* and its mutated variants. To indicate the duplication, the prefix ‘D-’ was added to the promoter variant name. (**B**) Relative EGFP fluorescence levels (RF) of D-P*_GTH1_* compared to native P*_GTH1_* and (**C**) RF of the ‘duplicated’ sorting (D-P_GS_*_n_*) and ‘duplicated’ single point mutation (D-P_GMutX_) variants compared to the ‘duplicated’ control D-P*_GTH1_*. Cells were cultivated in the 24-DWP screening format in either inducing (X, limiting glucose) or repressing (G, excess glycerol) conditions. Fluorescence was measured on a flow cytometer. Per variant at least four independent clones were analyzed, while for the D-P*_GTH1_* control at least six independent clones per screening were tested. Outliers were excluded from the analysis. Bars represent mean values and closed circles the calculated RF values of individual clones. Statistical analysis was done employing Student t-test (**P≤ 0.05; ***P≤ 0.005). The dotted line highlights the average expression level of the appropriate control (P*_GTH1_* for (B) and D-P*_GTH1_* for (C), respectively; set to 100% for each condition).

In a first engineering cycle, Prielhofer *et al.* (43) duplicated the main regulatory region of P*_GTH1_* yielding P*_GTH1_*-*D1240*, which enhanced EGFP expression strength by 1.6-fold without compromising repression on glycerol. Similar to P*_GTH1_*-*D1240*, we duplicated the −122 to −507 bp region of the sorting variants and selected single point mutations (Figure 4A). As the cloning method used for the duplication differed from the one employed for the creation of P*_GTH1_*-*D1240*, a new control (named D-P*_GTH1_*), which contained solely the duplication of the native promoter region without any mutations, was created and evaluated employing the established screening procedure. As expected (and similar to P*_GTH1_*-D1240), D-P*_GTH1_* showed a significant increase (1.8-fold) in induction strength and tighter repression properties than P*_GTH1_* (Figure 4B). In the following, the respective duplicated variants (named D-P_GS_*_n_* or D-P_GMutX_) were tested along D-P*_GTH1_* in inducing and repressing conditions as described before (Figure 4C). Indeed, all promoter variants outperformed D-P*_GTH1_* in terms of their induction strength. However, they also showed higher expression levels in glycerol excess condition. As mentioned above, D-P*_GTH1_* was slightly more repressed in glycerol excess than P*_GTH1_*, a feature that seemingly does not apply to the D-P_GS_*_n_* and D-P_GMutX_ variants.

The strongest variant in terms of inductions strength was again the variant in which point mutations MutB and MutP were combined. However, the expression level of this variant in repressing conditions was nearly 15-fold increased when compared to the D-P*_GTH1_* control. Taken together, the results indicate that duplication of the main regulatory region of P*_GTH1_* and its variants represents a very robust strategy for enhancing induction strength for this promoter family, while the effect on the repression characteristics is less consistent.

### Sliding window deletion analysis pinpoints the most crucial regulatory promoter regions

**Figure 5.**
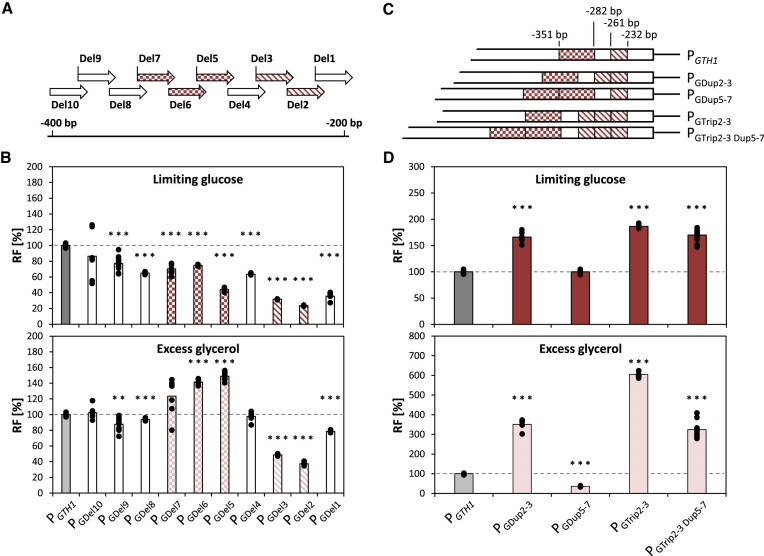
Sliding window deletions guide promoter engineering by segmental duplications. (**A**) Schematic representation of the segmental deletions introduced to the −200 to −400 bp region of P*_GTH1_*. Each deletion is 30 bp long and overlaps with the respective contiguous variants for 10 bps on each side. (**B**) Relative EGFP fluorescence levels (RF) of the different segmental deletion variants compared to P*_GTH1_*. (**C**) Schematic representation of the duplications or triplication of the key regions Del2-3 as well as Del5-7 and (**D**) respective relative EGFP fluorescence levels (RF) of the corresponding promoter variants compared to P*_GTH1_*. Cells for expression screenings were cultivated in the 24-DWP format in either inducing (limiting glucose) or repressing (excess glycerol) conditions. Per variant at least five independent clones were screened and compared to at least three independent cultivations of the P*_GTH1_* control strain. Outliers were excluded from the analysis. Bars represent mean values and closed circles the calculated RF values of individual clones. Fluorescence was measured by flow cytometry. Statistical analysis was done employing Student *t*-test (**P≤ 0.05; ***P≤ 0.005). The dotted line highlights the average expression level of the P*_GTH1_* control (set to 100% for each condition).

As discussed above, Prielhofer *et al.* (43) have previously identified the −400 to −200 bp region to contain the main regulatory features of the *GTH1* promoter, whose duplication robustly increases its induction strength. However, so far it remains unclear which of the approx. 200 bp confer the induction and repression properties of the promoter. Thus, we now deleted 30 bps segments of the TFBS-rich promoter sequence in a sliding window approach to understand their impact in more detail. The window size was chosen based on the fact that one DNA turn consist of 10.4 bp, with the aim to only minimally disturb the rotation of the DNA and architecture of neighbouring TFBS outside the targeted window. To cover the −200 to −400 bp region, a set of 10 deletion variants were constructed; each sharing 10 bps with the two neighbouring deletions (Figure 5A).

All deletion variants showed a decrease in induction strength in limiting glucose compared to P*_GTH1_*, while the impact on promoter repression varied significantly between the variants (Figure 5B), confirming the complex regulation of P*_GTH1_* and the relevance of the −200 to −400 bp region. However, with this strategy we were able to narrow down the important regions by identifying two groups of deletion variants with interesting expression patterns. P_GDel2_ and P_GDel3_ generally showed very low EGFP levels (approx. 30% relative to native P*_GTH1_* in limiting glucose, 40–50% on excess glycerol), indicating that the shared part contains a region with high importance for the global function of P*_GTH1_*. The second group identified was formed by P_GDel5_, P_GDel6_ and P_GDel7_, which were de-repressed by approx. 50% on glycerol, suggesting that the shared region is relevant for the repression properties of the promoter. Three of the deletions (P_GDel1_, P_GDel4_ and P_GDel8_) showed a decrease in induction strength but no significant change in terms of their repression properties. As they overlap with deletions Del2-3 and Del5-7, they were not chosen for further analysis. P_GDel10_ showed no significant effect on induction or repression. Together, the expression patterns obtained with these deletion variants allowed us to determine that the region that is shared between deletions 2 and 3 was more relevant for the general strength of the G*TH1* promoter, while the region that is shared in deletions 5, 6 and 7 was important for its repression. It is noteworthy that MutS as well as MutP fall into the region Del2-3, further highlighting its importance for induction strength.

### Segmental duplications of the key regions enhance the properties of the *GTH1* promoter

Since the promoter segments covered by the deletions Del2-3 and Del5-7 seem to have a key impact on the expression strength and repression properties of P*_GTH1_*, we decided to amplify the regions that were shared among them, respectively (Figure 5C). These promoter variants were called P_GDup2-3_ (duplicate positions –261 to –232 bp) and P_Gdup5-7_ (duplicate positions –351 to –282 bp) and tested in the established 24-DWP-screening format for their EGFP expression (Figure 5D). The variant P_GDup2-3_ showed nearly 60% higher EGFP levels in inducing conditions, however, repression on glycerol was significantly weaker compared to native P*_GTH1_* (3.5-fold higher expression levels on glycerol for P_GDup2-3_). On the other hand, duplication of region Del5-7 did not influence induction in limiting glucose but significantly impacted promoter repression (expression levels of P_GDup5-7_ on glycerol were approx. 60% lower). Taking these results into account, we next decided to triplicate region Del2-3 (P_GTrip2-3_) to determine if the positive effect on promoter activity could be even further amplified. Indeed, the induction strength of the promoter was further enhanced, however, at the same time EGFP levels on glycerol were up to 6.2-fold elevated when compared to P*_GTH1_*. Based on these results we decided to combine the mutations Trip2-3 with Dup5-7 and created P_GTrip2-3 Dup5-7,_ which indeed resulted in a variant whose expression levels in limiting glucose remained at the level of P_GTrip2-3_, while its EGFP levels in glycerol excess conditions remained below those of P_GDup2-3_ (Figure 5D).

### Combination of single point mutations and the sequential multiplication of key promoter segments enhances promoter induction strength synergistically

**Figure 6.**
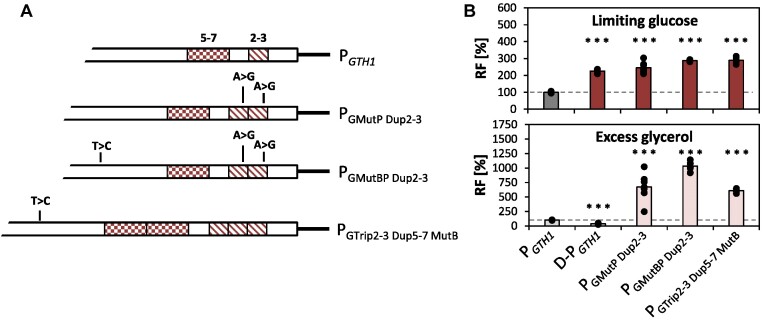
Combinatorial promoter variants. (**A**) Schematic representation of the promoter variants for which the segmental duplications and triplications as well as the most successful point mutations were combined. (**B**) Relative EGFP fluorescence levels (RF) of D-P*_GTH1_* as well as the different combinatorial variants compared to P*_GTH1_*. Per variant at least seven independent clones were compared to at least nine individual cultivations of the P*_GTH1_* control strain. Outliers were excluded from the analysis. Bars represent mean values and closed circles the calculated RF values of individual clones. Cells for expression screenings were cultivated in the 24-DWP format in either inducing (limiting glucose) or repressing (excess glycerol) conditions. Fluorescence was measured by flow cytometry. Statistical analysis was done employing Student t-test (***P≤ 0.005). The dotted line highlights the average expression level of the P*_GTH1_* control (set to 100% for each condition).

So far, the random mutagenesis approach has guided us to two single point mutations (MutB and MutP) which individually enhanced promoter activity under inducing conditions, and showed a positive synergistic effect in combination. On the other hand, the rational approach allowed us to more precisely locate those segments within the main regulatory region that are most relevant for the repression and induction properties of the *GTH1* promoter. Based on this information, we set out to combine the best performing variants from both approaches (Figure 6A). For this purpose, we first introduced MutB into P_GTrip2-3 Dup5-7_, resulting in variant P_GTrip2-3 Dup5-7 MutB_. We were not able to introduce MutP into this promoter variant as the location of this point mutation resides within region Del2-3. Thus, we duplicated region Del2-3 using MutP as a template, and further inserted MutB in this new variant, resulting in P_GMutP Dup2-3_ and the P_GMutBP Dup2-3_ variants, respectively.

To evaluate the expression behavior of these promoters we used both, P*_GTH1_* and D-P*_GTH1_*_,_ as references. Even though the new variants only contain segmental duplications, and not the duplication of the full 200 bps main regulatory region, their induction strength was comparable to or even higher than that of D-P*_GTH1_* (Figure 6B). P_GTrip2-3 Dup5-7 MutB_ and P_GMutBP Dup2-3_ resulted in 25% and 30% higher fluorescence when compared to D-P*_GTH1_*. Compared to the original *GTH1* promoter, the fluorescence in limiting-glucose increased 2.1- to 2.9-fold in the combinatorial promoter variants, being even stronger when MutB was present (Figure 6B). Introduction of MutB and/or MutP also further enhanced the induction strength compared to the segmental duplicated promoter variants (see Figure 6B compared to Figure 5D). As already observed before, repression on glycerol was much weaker than for the controls, with EGFP levels being 6.2- to 10-fold increased compared to P*_GTH1_*.

In conclusion, point mutations MutB and MutP and the segmental duplication act together synergistically in the combinatorial promoter variants, leading to two novel promoters exceeding expression strength of those mutated variants where the main regulatory region was fully duplicated.

### Differential repression characteristics of promoter variants in glucose and glycerol excess conditions point at distinct regulatory mechanisms

**Figure 7.**
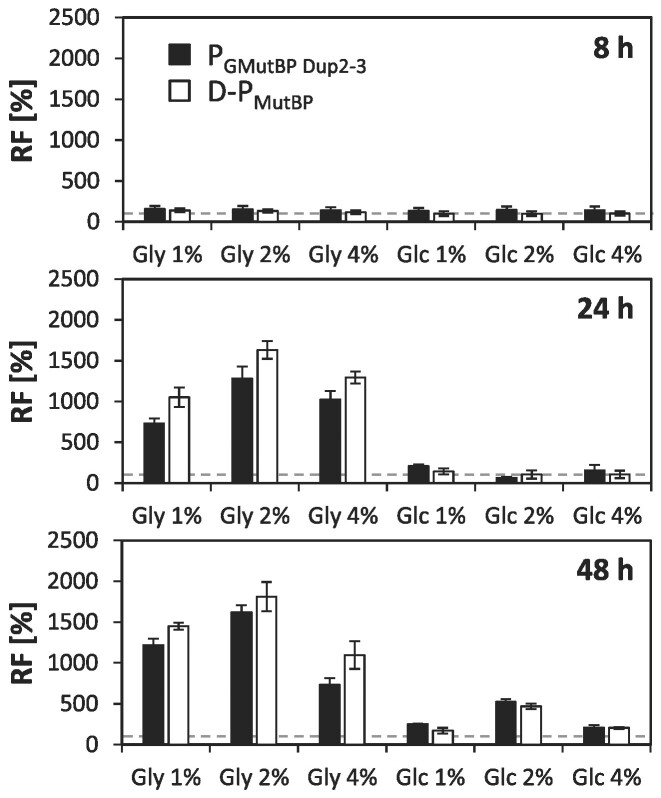
De-repression of promoter variants was evident in glycerol excess but not in glucose excess conditions. Relative EGFP fluorescence levels (RF) of the promoter variants P_GMutBP Dup2-3_ and P_GMutBP_ compared to D-P*_GTH1_*. Cells were cultivated in 24-deep-well-plates in minimal media supplemented with 1%, 2% or 4% glycerol (Gly) or glucose (Glc), respectively. Fluorescence levels were measured after 8, 24 and 48 h by flow cytometry. Per variant, four independent clones were analyzed. Bars represent mean values and error bars indicate standard deviation of the mean. The dotted line highlights the average expression level of the D-P*_GTH1_* control (set to 100% for each condition).

Native P*_GTH1_* is strongly repressed in glycerol excess. However, we observe even tighter repression in glucose excess conditions (own unpublished observation). Hence, the regulation of the promoter seems to differ between glycerol and glucose surplus and might therefore be affected differently by the various mutations. To evaluate how the partial loss in repression properties of some of the variants translates to glucose excess conditions and to test to which degree the carbon source concentration impacts this behavior, we selected two variants that showed a high loss in repression in excess glycerol, P_GMutBP Dup2-3_ and D-P_GMutBP_, and cultivated them in minimal media supplemented with 1%, 2% and 4% glycerol or glucose, respectively. Subsequently, EGFP levels were measured after 8, 24 and 48 h and compared to levels obtained for the D-P*_GTH1_* control strain (Figure 7).

After eight hours of cultivation, both promoter variants remained highly repressed in all tested conditions, with EGFP levels being similar or only slightly higher than those observed for D-P*_GTH1_*. However, after 24 and 48 h of cultivation, both variants showed a high degree of de-repression (7- to 18-fold) in all tested glycerol excess conditions. In contrast, in all the different tested glucose excess conditions EGFP levels of D-P_GMutBP_ and P_GMutBP Dup2-3_ remained mostly at the levels observed for D-P*_GTH1_*.

### The improved induction strength of the most promising promoter variants also translates into higher production of secreted recombinant proteins

**Figure 8.**
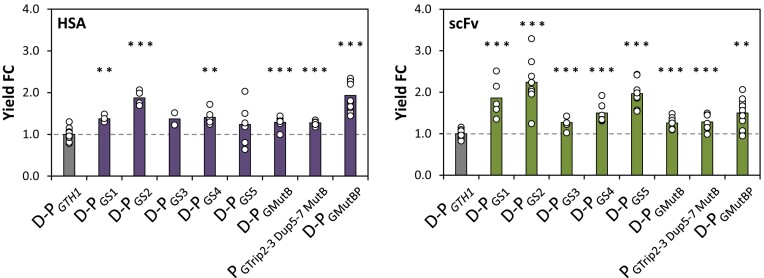
The novel promoter variants increase the production of secreted recombinant proteins in small scale screenings. Secreted protein yield fold-changes obtained for different promoter variants driving the expression of human serum albumin (HSA) or a single chain variable fragment (scFv) in comparison to the respective D-P*_GTH1_* control. Per promoter variant at least four (typically six or more) independent clones were compared against at least four independent cultivations of the D-P*_GTH1_* control strain. Outliers were excluded from the analysis. Bars represent mean values and open circles the calculated secreted protein yield of individual clones. Yields are based on extracellular recombinant protein titer and WCW measurements after 48 hours of incubation under limiting glucose (inducing) conditions in the 24-DWP format. Statistical analysis was done employing Student *t*-test (**P≤ 0.05; ***P≤ 0.005). The dotted line highlights the average yield of the respective D-P*_GTH1_* control (set to 100%).

So far, the promoter variants have only been employed for the expression of the intracellular reporter EGFP. However, in order to evaluate their potential for industrial use we tested the most interesting candidates for the expression of two different secreted recombinant proteins, namely human serum albumin (HSA) and a single chain variable fragment (scFv). At least four (typically six or more) independent clones per construct were analyzed in inducing conditions in small-scale screenings (applying glucose-limitation by a slow constant glucose release system) and compared to mean values of a representative clone of the appropriate D-P*_GTH1_* control strain. Obvious outliers that most likely derived from multiple integration of the expression cassette into the genome were not considered for the final analysis. For calculation of the product yield, secreted protein concentrations were measured in the supernatants and related to the wet cell weight at the end of the screening. Figure 8 shows the yield (μg_RP_ g^−1^ WCW) fold-change (FC) for the different secreted proteins compared to the D-P*_GTH1_* control strain. For both proteins, yields were clearly higher when using the novel promoter variants, albeit there was no explicit preference which sorting variant was the most beneficial in the screenings. Again, the variants containing MutB alone or in combination also exceed the product yields obtained when using D-P*_GTH1_*. Together, the data demonstrate that all novel promoter variants led to increased yields and therefore confirm that the positive effect observed for expression of the intracellular GFP reporter can indeed be translated to the production of secreted proteins that are of industrial interest.

### The novel promoter variants outperform D-P*_GTH1_* in glucose-based fed-batch cultivations

**Figure 9.**
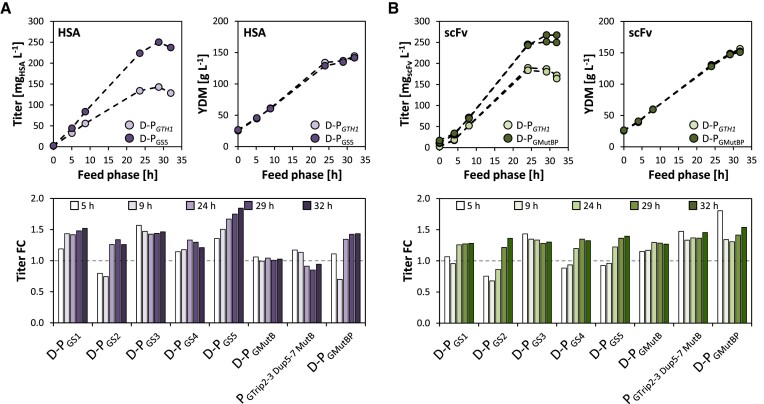
Secreted protein production by different promoter variants in glucose-based fed batch cultivations. Representative profiles of the measured (**A**) HSA and (**B**) scFv titers and biomass (YDM) concentrations throughout the glucose-limited fed-batch production phase as well as the titer fold-change (titer-FC) of the secreted protein calculated between the respective D-P*_GTH1_* control and the various promoter variants at the corresponding feed-phase sampling point. Secreted protein titers were measured in technical duplicates and corrected for the biomass concentration. If multiple fed-batch cultivations were conducted for a given variant, mean values were used to calculate the titer fold-change. The dotted horizontal line highlights the titers obtained for the respective D-P*_GTH1_* control.

To confirm that the various engineered variants also show increased activity in an industrial production process, glucose-limited fed batch cultivations were performed, employing the linear feeding profile previously optimized for P*_GTH1_-D1240* (43). The respective strains for fed-batch cultivations expressing the model proteins HSA or scFv under control of the promoter variants D-P_GS1-5_, D-P_GMutB_, D-P_GMutBP_ and P_GTrip2-3 Dup5-7 MutB_ were selected based on the results from small-scale screenings presented in Figure 8. Gene copy number (GCN) analysis by qPCR was performed to ensure that productivity levels were based on an equal expression cassette copy number (GCN was 1 for all selected clones and the control strains D-P*_GTH1_*-HSA and D-P*_GTH1_*-scFv). The respective control strain was cultivated along the different variants in each round of fed batch cultivation in one of the four parallel DASGiP bioreactor systems. As can be seen in the upper right panels of Figures 9A and B, there was no change in the biomass accumulation profiles.

The upper left panel of Figure 9A depicts representative profiles of the HSA titer throughout the production phase, while the titer fold-change (FC) calculated between the respective control and the various variants at the corresponding sampling points are shown in the lower panel of Figure 9A. All five D-P_GS_ variants as well as D-P_MutBP_ clearly outperformed D-P*_GTH1_*. D-P_GS5_ reached a nearly 2-fold higher HSA titer at the end of the production phase, while final titers for the other D-sorting variants were 20–50% increased. On the other hand, the variants D-P_GMutB_ and P_GTrip2-3 Dup5-7 MutB_ did not exceed titers reached with D-P*_GTH1_*-HSA, despite the fact that in small-scale screenings both variants outperformed D-P*_GTH1_* by approx. 30%. Notably, P_GTrip2-3 Dup5-7 MutB_, which has only the regions Del2-3 and Del5-7 multiplied, reached similar titers to D-P*_GTH1_* that contains a duplication of the whole TFBS-dense region.

As the performance of promoters can vary between different recombinant genes, the variants were tested for the expression of scFv in fed-batch cultivations as well. Similar to the results obtained for HSA, all D-P_GS1-5_ variants showed between 30% to 40% increased scFv-titers at the end of the fed-batch cultivation (lower panel of Figure 9B). In contrast to the previous fed-batch results for HSA, final scFv titers of the variants D-P_GMutB_ and P_GTrip2-3 Dup5-7 MutB_ exceeded the titers reached with the D-P*_GTH1_* control by 30% and 50%, respectively (Figure 9B). The highest scFv titer (around 55% increase) was reached by variant D-P_GMutBP_, the variant that also previously showed the highest activity in terms of EGFP expression in inducing conditions (Figure 4C). Interestingly, for most variants (especially D-P_GS4_, D-P_GS5_, D-P_GMutBP_ and to a lesser extend D-P_GS1_ and D-P_GS2_) higher final titers were predominantly based on higher specific production rates (q_P_) at the slower growth rates, which are obtained towards the end of the cultivation according to the applied feeding regime. The same trend was reflected in scFv transcript levels, which were for most variants higher 24 h into the feed phase than at the 5 h sampling point (Supplementary Figure S5 and Supplementary Table S5 in Supporting File 1).

As some of the variants showed high levels of de-repression in glycerol excess conditions in screenings (e.g. Figure 4), we took a closer look at the secreted recombinant protein concentrations measured at the end of glycerol batch phase (right before the glucose feed-phase was started; Supplementary Figure S6 in Supporting File 1). Indeed, those variants whose repression properties were affected the most according to previous EGFP measurements showed the highest concentration of secreted product at the end of the glycerol fed batch. Still, absolute values remained relatively low. For the variant P_GS3_, which was specifically selected based on its loss in repression and carries MutP, HSA and scFv concentrations were measured to be 10.9 and 9.6 mg l^−1^ at batch end, while their final titers reached more than 250 mg l^−1^ at the end of the fed batch. The titers for all other sorting variants at the end of batch phase were between 1 to 3.5 mg l^−1^ of recombinant protein, which is within the range of the respective D-P*_GTH1_* controls (0.9 mg l^−1^ HSA and 2.0 mg l^−1^ scFv, respectively). Taken together, even for those variants that showed the highest degree of de-repression in glycerol excess conditions, a maximum of 4% of the total product was produced during batch phase in the industrial production process. Thus, the observed de-repression is not critical when applying the novel promoter variants in industrial fed batch processes.

## Discussion

The *GTH1* promoter of *K. phaffii* is a strong and regulatable alternative to methanol-based systems (35), however, it was unknown which regions govern its repression in glucose and glycerol surplus or induction in limiting glucose conditions. Prielhofer et al. (43) showed that the region –472 to –178 bp of P*_GTH1_* conveys the main regulatory properties as well as the expression strength to the promoter, however, targeted deletion of certain TFBS (for e.g. F$ADR1, F$CSRE, F$RTG1) failed to enhance the expression strength (43).

To increase the expression strengths of P*_GTH1_* in a more unbiased approach, we used a random and a targeted strategy of nucleotide diversification. These strategies and their combination led us to find seven promoter variants that consistently result in higher titers than the original *GTH1* promoter (up to 3-fold higher). The novel variants also exceed the previously identified best variant P*_GTH1_*-*D1240* (now called D-P*_GTH1_*) containing a duplication of the main regulatory region (43) by 50–100% in glucose-limited screening and fed batch conditions. Furthermore, it was possible to obtain similar expression strength with shorter promoter variants (e.g. P_GMutBP Dup2-3_). Especially random mutagenesis and subsequent cell sorting, applying an adapted sorting strategy, proved to be most beneficial to obtain novel variants with improved induction and largely unaltered repression properties. Moreover, random mutagenesis also provided valuable insights to understand P*_GTH1_* regulation together with the segmental modifications. Similar to previous studies (e.g. reviewed in (15)), many of the randomly or rationally introduced point mutations led to lower expression, thereby verifying that even single nucleotide exchanges can alter the regulatory properties of a promoter. However, contrary to the previous work on the oxygen-responsive P*_DAN1_* promoter of *S. cerevisiae* (19), we were able to obtain promoter variants with improved induction properties by EP-PCR and subsequent cell sorting. By further scrutinizing some of the individual mutations present in the sorting variants, we were able to make two more important findings: First, there was a common denominator in the affected TF families, as they all regulate genes in response to nutrients (either carbon source or nitrogen availability). Second, we identified single point mutations that confers glycerol-mediated repression of P*_GTH1_*.

Specifically, CSRE and MIG1-TFBS could be pinpointed as the major regulatory sites of the promoter, and reverse engineering led to improved induction capacities of P*_GTH1_* and D-P*_GTH1_*. Our results agree with the hypothesis that transcriptional activity in eukaryotes is enhancer limited (10), and show that enhancing the number of CSRE is a beneficial strategy to strengthen promoter activity in glucose limitation. Strikingly, not the presence or abundance of these TFBS proved to be the most important regulatory element but rather there was a clear site dependency, which could only be revealed through the random mutagenesis approach and subsequent reverse engineering of the promoter. Furthermore, our results indicate that for successful promoter engineering it is important to only modify the targeted TFBS, while avoiding the creation or deletion of additional TFBS.

Using our combinatorial promoter engineering approach, it was possible to attribute expression strength as well as glycerol-mediated repression to two distinct TFBS, one CSRE and one MIG-TFBS. The deletion of the MIG binding site in P_GS3_, as well as in P_GMutP_ and PG_MutBP_, has a clear impact on the repression properties of the promoter variants. It seems that this specific TFBS is conferring a high degree of repression on glycerol, which can be traced back to the action of the repressor Mig. *K. phaffii* Mig1 homologs Mig1-1 and Mig1-2 have been reported to repress P*_AOX1_* on glycerol, while their deletion had no effect on the expression of *AOX1* in glucose surplus (40,52). Simultaneous deletion of the Mig1 homologs did not only de-repress *AOX1* expression in glycerol excess conditions, but led also to higher expression levels of many genes involved in methanol utilization and central carbon metabolism, suggesting a more general role of Mig1-1 and Mig1-2 in glycerol repression in *K. phaffii*.

Our data indicate that Mig1-1 and Mig1-2 are also involved in the repression of P*_GTH1_* in glycerol excess, while – consistent with previous observations – glucose repression seems to be governed by other factors. These results highlight the importance of a fine-tuned glucose import regulation in *K. phaffii*.

Interestingly, deletion of the glycerol-responsive MIG-TFBS in P*_GTH1_* also led to higher expression levels on limiting glucose. The positive impact of deleting one MIG TFBS on expression strength in glucose limiting induction conditions is less obvious, but might be explained through the finding that Mig1-2 represses the expression of one of the genes encoding the CSRE binding transcriptional activators (the Sip4 homolog Cat8-2) on both glycerol and glucose (53). Additionally, the deletion of the MIG site in MutP might also uncover a F$CSRE that is normally inaccessible due to steric hindrance (see scheme in Supplementary Figure S4 in Supporting File 1). In *S. cerevisiae*, it was also reported that the Mig1/2 repressors can interfere with binding of the Cat8/Sip4 activator proteins to the CSRE (54,55).

Even though some variants lacking certain MIG-TFBS show higher de-repression on glycerol in small scale screenings, promoter repression throughout glycerol batch phase in bioreactor conditions was still sufficient. Alternatively, these two novel promoter variants could be employed for a different expression strategy, as they are tightly repressed in glucose excess, show medium expression in glycerol excess and high induction in limiting glucose.

## Conclusions

Through a nested interplay of random and rational promoter engineering, we advanced our understanding of promoter regulation and were able to identify a library of novel P*_GTH1_* promoter variants that exhibit increased expression strengths in inducing conditions. We provide evidence that nucleotide diversification along with an adapted tailor-made sorting strategy is a suitable means to obtain superior variants of inducible promoters such as P*_GTH1_*. Furthermore, through this integrated reverse engineering approach, it was possible to identify single nucleotide positions and correspondingly affected binding sites of transcriptional regulators that contribute to the high expression and distinguish between glycerol- and glucose-mediated repression of the native *GTH1* promoter.

Finally, after initial characterization of the promoter variants with the intracellular reporter GFP, the improved variants were verified to increase also the production of two secreted proteins in industrially relevant fed batch conditions. The universal applicability of the novel promoter variants makes them an important part of the toolbox to advance both recombinant protein production and metabolic engineering of *K. phaffii*. In addition to demonstrating the advantages and application potentials of the newly generated promoter variants for biotechnological production processes, several interesting discoveries were made throughout this work. Most importantly, our results provide answers to the intriguing and challenging question how mutations of only a few nucleotides in DNA regulatory elements can impact on the transcriptional activity on different carbon sources.

## Supplementary Material

gkad752_Supplemental_File

## Data Availability

All data are included in the manuscript or its supplements.

## Notes

Present address: Roland Prielhofer, Phoenestra GmbH, Linz, Austria.
