# Supplementary material for: Systematic sequence engineering enhances the induction strength of the glucose-regulated GTH1 promoter of Komagataella phaffii
Source: Nucleic Acids Res. 2023 Oct 4;51(20):11358–74. doi: 10.1093/nar/gkad752 (PMC10639056; doi:10.1093/nar/gkad752)
Supplement: gkad752_Supplemental_File [file gkad752_supplemental_file.pdf]

Systematic sequence engineering enhances the induction strength of the glucose-regulated *GTH1* promoter of *Komagataella phaffii*

Mirelle Flores-Villegas*, Corinna Rebnegger*, Viktoria Kowarz, Roland Prielhofer[$], Diethard Mattanovich, Brigitte Gasser

*equally contributing

[1] CD-Laboratory for Growth-decoupled Protein Production in Yeast at Department of Biotechnology, University of Natural Resources and Life Sciences (BOKU), Vienna, Austria

[2] University of Natural Resources and Life Sciences Vienna (BOKU), Department of Biotechnology, Institute of Microbiology and Microbial Biotechnology, Muthgasse 18, 1190 Vienna, Austria

[3] ACIB GmbH, Muthgasse 11, 1190 Vienna, Austria

[$] Present address: Phoenestra GmbH, Linz, Austria

This file includes the following:

Supplementary Tables S1, S2, S3, S4, S5

Supplementary Figures S1, S2, S3

Table S1: List of primers used in this study.

Name	Product/Purpose	Type	Sequence
**PE1**	EP-PCR P_{GTH1}	Fw	TAGACTTCAAGATCTCAGGGATTCCCA
**PE2**	EP-PCR P_{GTH1}	Bw	CAACTAATCCAATAAATTGGCTCATCTTG
**PE3**	variant sequencing	Fw	TACGGCGCCGCCATATTG
**PE4**	variant sequencing	Bw	CTGAACTTGTGGCCGTTTAC
**PE5**	variant + EGFP amplification	Fw	GTAGTCGAAGACCCGGAGCCAAACATTTGCTCCCCCTAG
**PE6**	variant + EGFP amplification	Bw	GTCGTAGAAGACATAGCGAGCTTGCAAATTAAAGCCTTCG
**PE7**	MutA	Fw	GGTATTCcGATATGTTTTTCCTG
**PE8**	MutA	Bw	ACATATCgGAATACCAAATAGTG
**PE9**	MutB	Fw	CCACTATTcGGTATTCTGATATG
**PE10**	MutB	Bw	CATATCAGAATACCgAATAGTGG
**PE11**	MutC	Fw	GTCCGGATAAaAGAATTTTG
**PE12**	MutC	Bw	CAAAATTCTtTTATCCGGAC
**PE13**	MutD	Fw	CTTGTCCGGATgAGAGAATTTTG
**PE14**	MutD	Bw	CAAAATTCTCTcATCCGGACAAG
**PE15**	MutP	Fw	GGAAGTGCCCAGAgTTTTC
**PE16**	MutP	Bw	GTAATCCCCGGAAAAcTCTG
**PE17**	MutQ	Fw	GAATTTTCCGGGaATTACGGATAATAC
**PE18**	MutQ	Bw	GTATTATCCGTAATtCCCGGAAAATTC
**PE19**	MutR	Fw	CCGGTTATaACCCCACC
**PE20**	MutR	Bw	GGTGGGGTtATAACCGG
**PE21**	MutS	Fw	GTTATTACCCCAaCTGGAAGTG
**PE22**	MutS	Bw	CACTTCCAGtTGGGGTAATAAC
**PE23**	MutT	Fw	GTTATTACCCCACCgGGAAGTG
**PE24**	MutT	Bw	CACTTCCcGGTGGGGTAATAAC
**PE25**	MutU	Fw	GAAGTGCCCgGAATTTTC
**PE26**	MutU	Bw	GAAAATTCcGGGCACTTC
**PE27**	Duplication - Fragment 1	BW	GTAGTCGAAGACCCAGCCTGTTGTACTTCAGCTAA
**PE28**	Duplication - Fragment 2	Fw	GTCGTAGAAGACATGGCTAATTCCCTGAAAAAACT
**PE29**	Duplication - Fragment 2	BW	GTCGTAGAAGACATAATTGGCTCATCTTGTTTAT
**PE30**	Duplication - Fragment 3	Fw	GTCGTAGAAGACATAATTCCCTGAAAAAACTGCA
**PE31**	Del1	Fw	GTAGTCGGTCTCCATTTGATTAATTAATACGCCAAGTC
**PE32**	Del1	Bw	GTAGTCGGTCTCCAAATTCTGGGCACTTCCAGGT
**PE33**	Del2	Fw	GTAGTCGGTCTCTCCCAACGGATAATACGGTGG
**PE34**	Del2	Bw	GTAGTCGGTCTCCTGGGGTAATAACCGGAAAAATATG
**PE35**	Del3	Fw	GTAGTCGGTCTCCCATACCCAGAATTTTCCGGGG
**PE36**	Del3	Bw	GTAGTCGGTCTCCTATGGAGCAGGCGTCCATTTA
**PE37**	Del4	Fw	GTAGTCGGTCTCCGGATTATTACCCCACCTGGAAGTG
**PE38**	Del4	Bw	GTAGTCGGTCTCCATCCGAACGGATAATCAAACA
**PE39**	Del5	Fw	GTAGTCGGTCTCCTTTTCTGCTCCATATTTTTCCGGT
**PE40**	Del5	Bw	GTAGTCGGTCTCCAAAATTCTCTTATCCGGACAAGA
**PE41**	Del6	Fw	GTAGTCGGTCTCCGTCTCCGTTCGGATAAATGGAC
**PE42**	Del6	Bw	GTAGTCGGTCTCCAGACCCCGTTGGAAATTAATT
**PE43**	Del7	Fw	GTAGTCGGTCTCCACAAAGAGAATTTTGTTTGATTATCCGTTC

**PE44**	Del7	Bw	GTAGTCGGTCTCCTTGTCACGAAAACGGGGT
**PE45**	Del8	Fw	GTAGTCGGTCTCCTTGAAACGGGGTCTTGTCCGGA
**PE46**	Del8	Bw	GTAGTCGGTCTCCTCAAAAAAAAAAAAAAATAGCGGAGATTCAGG
**PE47**	De9	Fw	GTAGTCGGTCTCCTCCGTTCGTGACAAATTAATTTCCAACG
**PE48**	Del9	Bw	GTAGTCGGTCTCCCGGAGATTCAGGTTTTAGATTAGAG
**PE49**	Del10	Fw	GTAGTCGGTCTCCTCTATTTTTTTTGATGACCCCGTTTTC
**PE50**	Del10	Bw	GTAGTCGGTCTCCTAGAGTTTTGATGCATATCAGG
**PE51**	Dup2-3	Fw	GTAGTCGAAGACCCTTTATTACCCCACCTGGAAGTG
**PE52**	Dup2-3	Bw	GTAGTCGAAGACCCTAAAATTCTGGGCACTTCCAGG
**PE53**	Dup5-7	Fw	GTAGTCGAAGACCCGCATTAATTTCCAACGGGGTC
**PE54**	Dup5-7	Bw	GTAGTCGAAGACCCATGCGTCCATTTATCCGAACG
**PE55**	*ACT1* qPCR	Fw	CCTGAGGCTTTGTTCCACCCATCT
**PE56**	*ACT1* qPCR	Bw	GGAACATAGTAGTACCACCGGACATAACGA
**PE57**	EGFP qPCR	Fw	TCGCCGACCACTACCAGCAGAA
**PE58**	EGFP qPCR	Bw	ACCATGTGATCGCGCTTCTCGTT
**PE59**	EGFP qPCR v2	Fw	CCCGCGCCGAGGTGAAGT
**PE60**	EGFP qPCR v2	Bw	TTCTTCTGCTTGTCGGCCATGATATAG
**PE61**	HSA qPCR	Fw	AGAGAGACAGATCAAGAAGCAGAC
**PE62**	HSA qPCR	Bw	CCAACTTCTTACCCTCTTCAGCG
**PE63**	α-MF leader qPCR (for scFv)	Fw	GCTGCCCCTGTTAACACT
**PE64**	α-MF leader qPCR (for scFv)	Bw	CCCTCTTCCTTAGCAGCAA
**PE65**	scFv qPCR	Fw	AAGCCTGGTAAGCCTCCAAAGT
**PE66**	scFv qPCR	Bw	TCCTCAGCTTGAACACCACCAAT

Table S2. List of putative TFBS from Matinspector of the targeted region for EP-PCR

Matrix Family	Matrix	Start position	End position	Strand	Sequence
**F$RRPE**	F$STB3.01	-488	-504	-	tgcagtttTTTCaggga
**F$MGCM**	F$RGT1.02	-426	-442	-	atatcAGGAaaaacata
**F$GATA**	F$GZF3.01	-420	-434	+	tcctGATAtgcatca
**F$PHD1**	F$PHD1.01	-418	-430	+	gataTGCAtcaaa
**F$YMAT**	F$MATA1.01	-417	-429	-	ttttGATGcatat
**F$ICGG**	F$CHA4.01	-388	-408	+	taaaacctgaatctCCGCtat
**F$MGCM**	F$YRR1.01	-387	-403	-	aatagCGGAgattcagg
**F$RDR1**	F$RDR1.01	-389	-399	-	tagCGGAgatt
**F$RFXP**	F$RFX1.02	-352	-366	-	ttgtcacgaAAACgg
**F$YMCB**	F$SWI4.01	-352	-364	-	ttgtcaCGAAaac
**F$RRPE**	F$STB3.01	-345	-361	-	aattaattTGTCacgaa
**F$TALE**	F$CUP9.01	-347	-359	-	ttaattTGTCacg
**F$BZIP**	F$YAP1.02	-341	-361	-	tggaaattaatttGTCAcgaa
**F$HOMD**	F$YOX1.01	-344	-358	-	aaattAATTtgtcac
**F$HOMD**	F$YOX1.01	-343	-357	+	tgacaAATTaatttc
**F$ICGG**	F$TEA1.01	-337	-357	+	tgacaaaTTAAtttccaacgg
**F$MGCM**	F$YRR1.01	-336	-352	-	cccgtTGGAaattaatt
**F$ASG1**	F$ASG1.01	-324	-340	-	tCCGGacaagaccccgt
**F$MGCM**	F$RGT1.02	-321	-337	-	ttatcCGGAcaagaccc

**F$RDR1**	F$RDR1.01	-320	-330	+	gtcCGGAtaag
**F$MGCM**	F$RGT1.02	-316	-332	+	ttgtcCGGAtaagagaa
**F$GATA**	F$GATA.01	-315	-329	+	tccgGATAagagaat
**F$PRES**	F$STE12.01	-303	-315	-	taatcaAACAaaa
**F$GATA**	F$GAT1.01	-297	-311	-	aacggATAAtcaaac
**F$MGCM**	F$RGT1.02	-294	-310	-	ccgaaCGGAtaatcaaa
**O$MTEN**	O$DMTE.01	-290	-310	-	ttatccgAACGgataatcaaa
**F$YORE**	F$OAF1.01	-283	-307	-	cgtccatttaTCCGaacggataatc
**F$RDR1**	F$RDR1.01	-289	-299	+	gttCGGAtaaa
**F$MGCM**	F$RGT1.02	-285	-301	+	ccgttCGGAtaaatgga
**F$CSRE**	F$SIP4.01	-285	-299	-	tCCATttatccgaac
**F$YGAL**	F$GAL4.01	-277	-301	-	agcaggcgtccatttatCCGAacgg
**F$YGAL**	F$LAC9.01	-275	-299	+	gttCGGAtaaatggacgcctgctcc
**F$CSRE**	F$CSRE.01	-261	-275	+	catattttTCCGgtt
**F$FBAS**	F$LEU3.02	-260	-276	-	taaCCGGaaaaatatgg
**F$MGCM**	F$RGT1.01	-259	-275	-	ataacCGGAaaaatatg
**F$ICGG**	F$TEA1.01	-249	-269	-	aggtgggGTAAtaaccggaaa
**F$RDNA**	F$REB1.02	-250	-262	+	ttaTTACcccacc
**F$YADR**	F$ADR1.01	-250	-258	+	taCCCCacc
**F$YMCM**	F$MCM1.02	-244	-260	-	cTTCCaggtggggtaat
**F$YMIG**	F$MIG1.01	-242	-260	-	cacttccaggtGGGGtaat
**F$MGCM**	F$RGT1.02	-223	-239	-	atcccCGGAaaattctg
**F$YMIG**	F$MIG1.01	-221	-239	+	cagaattttccGGGGatta
**F$PDRE**	F$PDRE.01	-224	-232	-	TCCCcggaa
**F$PDRE**	F$PDRE.01	-223	-231	+	TCCGgggat
**F$ARPU**	F$PPR1.01	-215	-231	+	tccggggattaCGGAta
**F$CYTO**	F$HAP1.01	-216	-230	+	ccggggatTACGgat
**F$ICGG**	F$TEA1.01	-213	-233	-	attatccGTAAtccccggaaa
**F$YQA1**	F$QA1F.01	-208	-228	+	ggggattacggaTAATacggt
**F$MGCM**	F$RGT1.02	-209	-225	+	gattaCGGAtaatacgg
**F$CYTO**	F$HAP1.01	-207	-221	+	acggataaTACGgtg
**F$BZIP**	F$CIN5.01	-188	-208	+	tggtctggattaatTAATacg
**F$HOMD**	F$YOX1.02	-189	-203	-	gtattaATTAatcca
**F$HOMD**	F$YOX1.02	-188	-202	+	ggattaATTAatacg
**F$BZIP**	F$CIN5.01	-183	-203	-	cttggcgtattaatTAATcca
**F$YABF**	F$ABF1.04	-184	-202	+	ggATTAattaatacgccaa
**F$PHRR**	F$RIM101.01	-176	-192	+	atacGCCAagtcttaca
**F$PRES**	F$STE12.01	-163	-175	-	gactgcAACAaaa
**F$FKHD**	F$FKH2.01	-132	-148	+	gcaataaTAAAcaagat

Table S3: Effect of transformation conditions and FACS gate positioning on multicopy integration frequency. Clones were pre-screened for multicopy integration of the EGFP expression cassette by Sanger sequencing and confirmed by qPCR.

	Number of analysed clones	Number of multicopy clones	Multicopy frequency
K. phaffii **Library 1**	48	8	16.7%
K. phaffii **Library 2**	56	0	n.a.
**Sort 1 "Top 1%" (excluded population)**	30	22	73.3%
**Sort 1 "Good 20%" (included population)**	28	3	10.7%

Table S4: Additional information for the respective point mutations of the five promoter variants isolated from the sorting procedure for which a higher induction strength could be confirmed.

Variant	Total number of mutations	Number of transitions	Number of mutations inside a TFBS
P_{GS1}	7	6	2
P_{GS2}	6	2	6
P_{GS3}	5	5	4
P_{GS4}	3	2	1
P_{GS5}	8	4	7
**Total**	**29**	**19**	**20**

Table S5. Relative scFv transcript level fold changes (FC) after 5 h and 24 h into the bioreactor cultivation feed phase. All strains were confirmed by qPCR to carry a single scFv expression unit.

Strain	Transcript level FC 5 h	Transcript level FC 24 h
D-P_{GTH1} scFv*	1.00	1.00
D-P_{GS1} scFv	1.35	1.78
D-P_{GS2} scFv	1.25	2.21
D-P_{GS3} scFv	1.35	1.74
D-P_{GS4} scFv	1.34	1.46
D-P_{GS5} scFv #1	1.26	1.54
D-P_{GS5} scFv #2	1.36	1.73
D-P_{GMutB} scFv	1.67	3.11
$PG_{Trip2\text{-}3\ Dup5\text{-}7\ MutB}$ scFv	1.60	1.82
D-P_{GMutBP} scFv #1	2.29	2.14
D-P_{GMutBP} scFv #2	1.26	1.90

*set to 1.00.

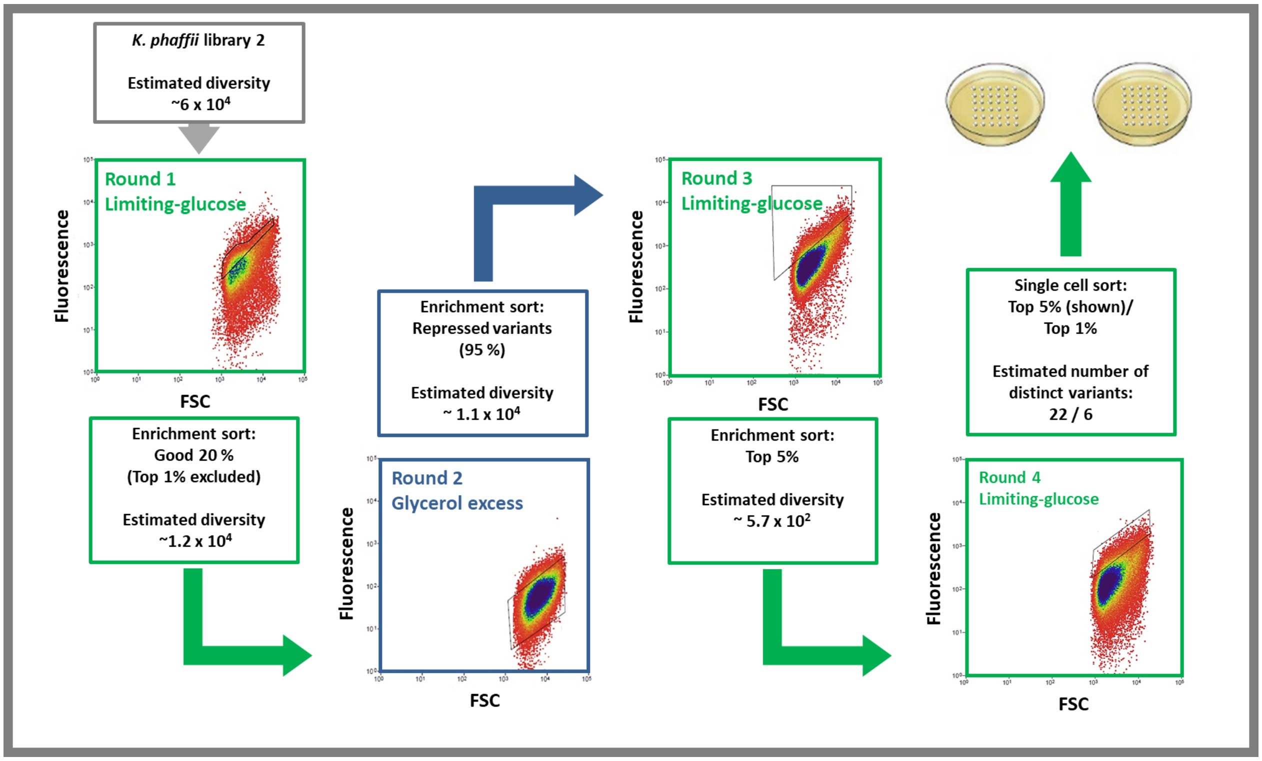

Figure S1. Main sorting workflow: $P_{GTH1\text{-mut}}$ variants with improved induction strength were isolated by subjecting *K. phaffii* library 2 to four rounds of fluorescence activated cell sorting (FACS). Prior to every FACS round the cells were either grown in inducing (limiting glucose; X) or repressing (glycerol excess; G) conditions. The estimated library diversity after gating is indicated at every step. Sorting variants P_{GS1}, P_{GS2}, P_{GS4} and P_{GS5} were derived from this procedure.

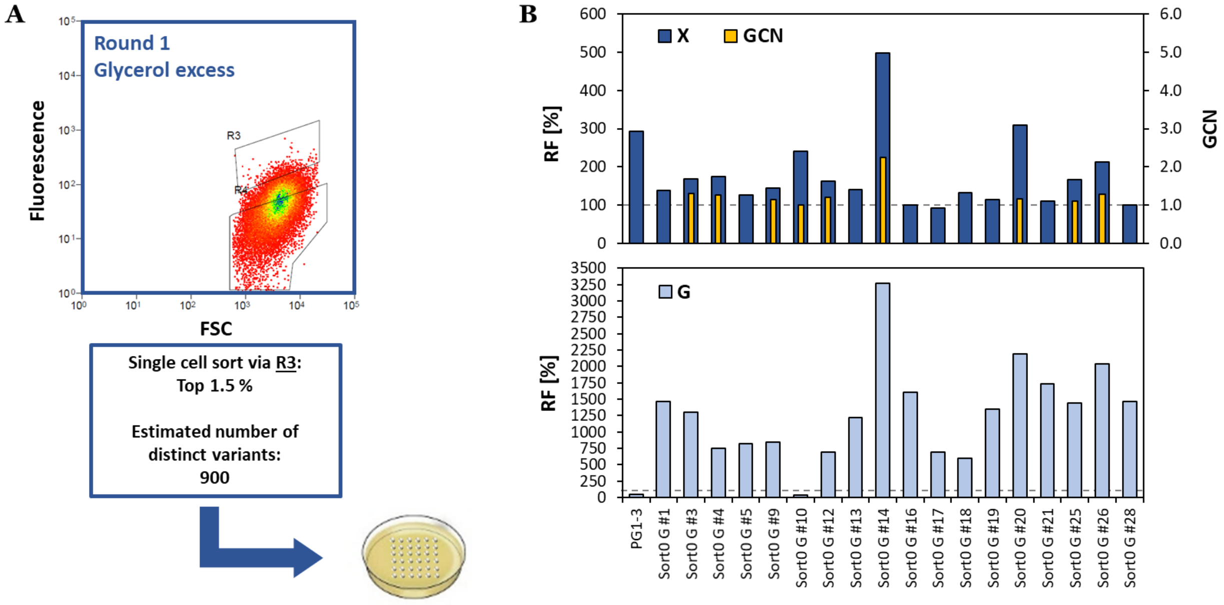

Figure S2. A) Isolation of variants from *K. phaffii* library 2 which showed high fluorescence in repressing (excess glycerol) conditions by FACS and **B)** EGFP expression screenings of these variants in limiting glucose (X) and excess glycerol (G) conditions. Bars represent relative fluorescence levels (RF) compared to the native *GTH1* promoter as well as EGFP copy number (GCN) as determined by qPCR. Cells were grown in the 24-DWP format and EGFP levels measured on a flow cytometer. Relative fluorescence levels were calculated by normalizing to the P_{GTH1} control. Sorting variants P_{GS3} was derived from this procedure (Sort0 G#20).

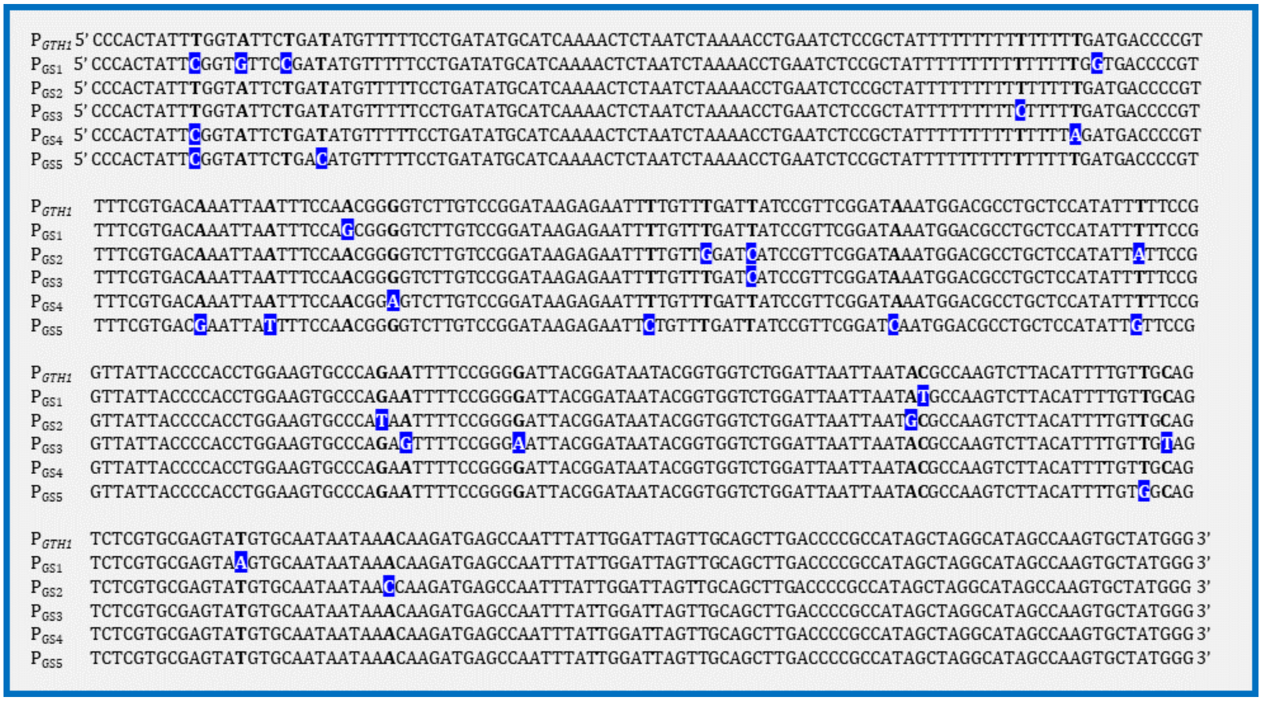

Figure S3. Sequence alignment of the mutated promoter region of the five variants isolated after the random mutagenesis process through FACS for which an increased induction strength could be confirmed following re-cloning into Golden*Pi*CS. The depicted sequence starts at position –472 bp and ends at position – 76bp of the *GTH1* promoter relative to the eGFP start codon.

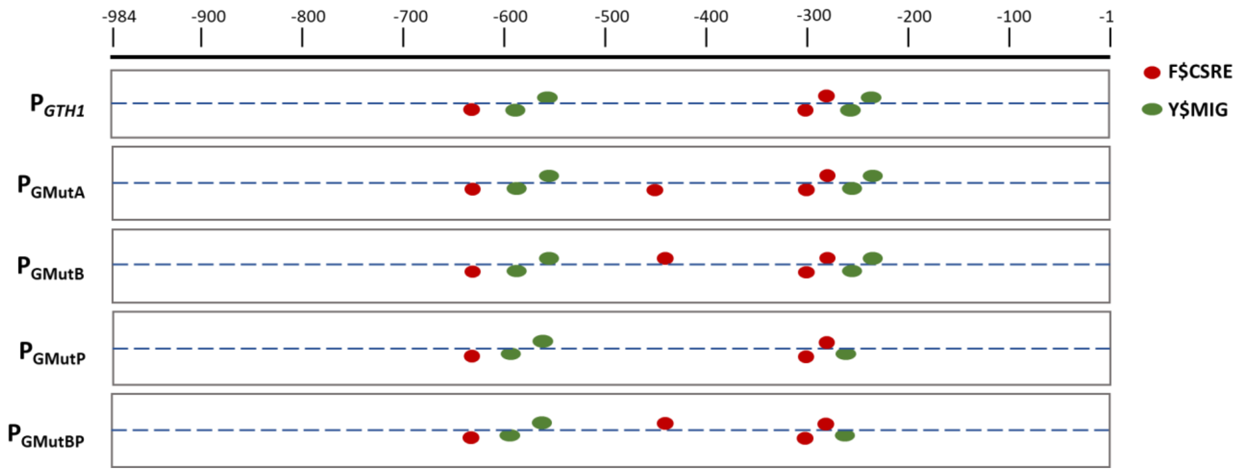

Figure S4. Schematic illustration of TFBS of the native *GTH1* promoter and the relevant mutants analyzed *in-silico* by MatInspector. Red ellipses represent F$CSRE and green ellipses Y$MIG. P_{GMutA} and P_{GMutB} gain one F$CSRE at the respective opposite strand while one Y$MIG site is lost in the proximity to another F$CSRE in P_{GMutP}.

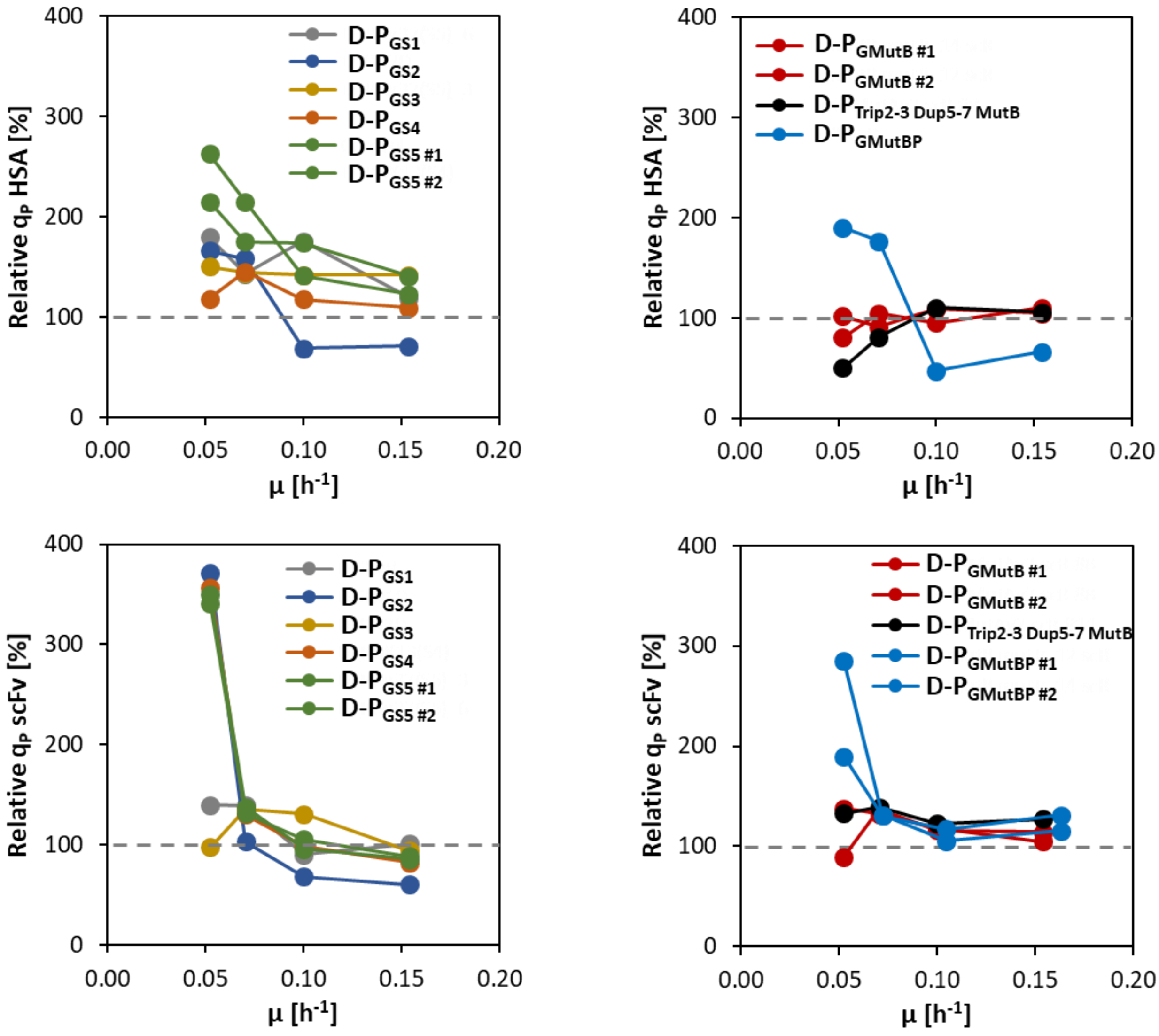

Figure S5. Relative specific production rates (q_P) of HSA and scFv for different promoter variants in relation to the specific growth rate (μ) throughout the first 29 hours of the fed-batch feed phase. The specific productivity of the various variants was calculated between the respective sampling points and normalized by comparing to the corresponding q_P-value of the respective D-P*GTH1* control strain.

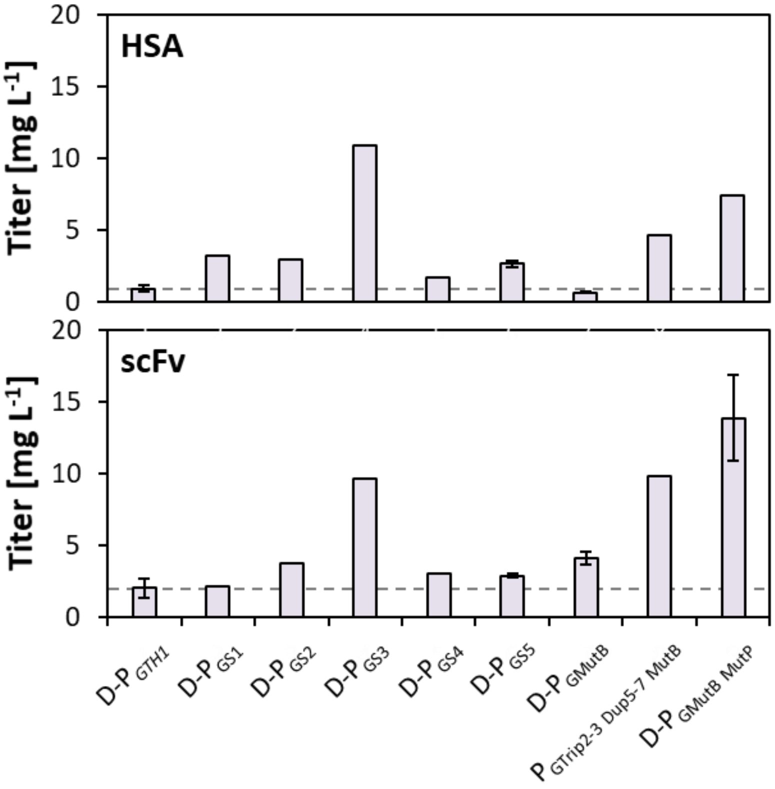

Figure S6. Secreted protein titer after glycerol batch phase. Bars represent HSA and scFv titer measurements after the glycerol batch phase was completed. If multiple fed-batch cultivations were conducted for a given variant, the mean value is depicted. Error bars represent the standard deviation of the mean calculated across independent fed-batch cultivations. The dotted horizontal line highlights the titers obtained for the respective D-P_{GTH1} control.
